# Cell-free DNA topology depends on its subcellular and cellular origins in cancer

**DOI:** 10.1172/jci.insight.159590

**Published:** 2022-10-24

**Authors:** Ethan Z. Malkin, Steven De Michino, Meghan Lambie, Rita Gill, Zhen Zhao, Ariana Rostami, Andrea Arruda, Mark D. Minden, Scott V. Bratman

**Affiliations:** 1 Department of Medical Biophysics, Temerty Faculty of Medicine, University of Toronto, Toronto, Ontario, Canada.; 2 Princess Margaret Cancer Centre, University Health Network, Toronto, Ontario, Canada.; 3 Department of Radiation Oncology, Temerty Faculty of Medicine, University of Toronto, Toronto, Ontario, Canada.

**Keywords:** Cell Biology, Oncology, Cancer, Diagnostics, Mitochondria

## Abstract

Cancer cells release large quantities of cell-free DNA (cfDNA) into the surrounding tissue and circulation. As cfDNA is a common source of biomarkers for liquid biopsy and has been implicated as a functional mediator for intercellular communication, fundamental characterization of cfDNA topology has widespread biological and clinical ramifications. Whether the topology of cfDNA is such that it exists predominantly in membrane-bound extracellular vesicles (EVs) or in nonvesicular DNA-protein complexes remains poorly understood. Here, we employed a DNA-targeted approach to comprehensively assess total cfDNA topology in cancer. Using preclinical models and patient samples, we demonstrate that nuclear cfDNA is predominantly associated with nucleosomal particles and not EVs, while a substantial subset of mitochondrial cfDNA is membrane protected and disproportionately derived from nontumor cells. In addition, discrimination between membrane-protected and accessible mitochondrial cfDNA added diagnostic and prognostic value in a cohort of head and neck cancer patients. Our results support a revised model for cfDNA topology in cancer. Due to its abundance, nuclear cfDNA within nucleosomal particles is the most compelling liquid biopsy substrate, while EV-bound and accessible mitochondrial cfDNA represent distinct reservoirs of potential cancer biomarkers whose structural conformations may also influence their extracellular stability and propensity for uptake by recipient cells.

## Graphical abstract

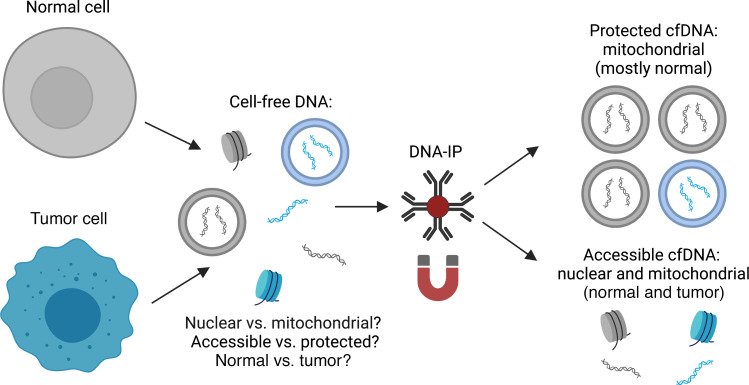

## Introduction

Cell-free DNA (cfDNA) exists in the extracellular compartment of all tissues and within bodily fluids. During tissue homeostasis (1), physiologic cellular turnover produces cfDNA via the cell death processes of apoptosis and necrosis (2–4). Interestingly, DNA can be actively secreted by live cells into the extracellular space (3, 5). In cancer, tumor-derived cfDNA can remain within the tumor microenvironment or enter the circulation. In both settings, cfDNA has been implicated in mediating various functional and pathophysiological processes (6, 7). Additionally, circulating tumor-derived cfDNA has rapidly emerged as a key clinical biomarker in liquid biopsy applications (8, 9). While cfDNA is promising as both a bioactive entity and clinical tool, its heterogeneous composition has presented a challenge to understanding its structural and biophysical properties.

Tumor-derived cfDNA can exist in many forms depending on its subcellular origin and mechanism of release. In apoptotic cells, caspase-activated DNases cleave histone-bound nuclear DNA (nDNA) into mono- and oligo-nucleosomes, which are released into the extracellular space (10, 11). Live cells may also secrete histone-bound nDNA (3, 12). Secreted mitochondrial DNA (mtDNA) can exist as intact mitochondrial nucleoid particles or smaller protein-bound fragments (13). In addition, a novel class of nucleic acid-protein complexes termed extracellular particles was recently described, though their composition and the characteristics of their DNA contents remain largely unknown (14–16).

Tumor cells might also release cfDNA via larger, membrane-bound entities. Extracellular vesicles (EVs) are membranous parcels that carry proteins and nucleic acids reflecting the cytosolic composition of their cell of origin. EVs can originate from the plasma membrane or endosomal system, with distinct EV populations being defined by their biophysical properties and specific protein markers (17, 18). For example, many EVs carry flotillin1, HSP90, and syntenin1, while others harbor the transmembrane proteins CD9, CD63, and CD81 (19–21).

Both nDNA and mtDNA have been implicated as cargos of EVs. As such, EV-associated DNA has emerged as a potentially novel liquid biopsy substrate and functional mediator of intercellular communication (18, 22–24). However, the absence of standardized isolation and analysis techniques has resulted in conflicting findings in the literature and lack of consensus regarding EV composition and contents (17, 25, 26). In fact, the existence of EV-associated DNA is far from certain: several recent studies have cast doubt on the presence of substantial quantities of DNA within EVs (12, 27, 28). These conflicting reports of EV-associated DNA have highlighted a lack of knowledge of tumor-derived cfDNA topology. Careful elucidation of the biophysical properties of cfDNA is crucial to understanding its functional roles and clinical implications.

Here, we sought to conduct a comprehensive assessment of total cfDNA released in the context of cancer. We developed a DNA-targeted immunoprecipitation (DNA-IP) approach coupled with various downstream assays to characterize the topology of cfDNA from both preclinical models and human samples. We found that nDNA is rarely associated with EVs. Conversely, a portion of mtDNA is protected within membranous structures. Surprisingly, we also found that within tumor-bearing individuals, the majority of membrane-protected cf-mtDNA was actually derived from nontumor cells, while the accessible cf-mtDNA subset showed promise as a diagnostic and prognostic biomarker. These findings contribute to our understanding of subcellular and cellular origins of cfDNA and have important implications for ongoing study of EV-associated DNA as a cancer biomarker and functional mediator.

## Results

### The vast majority of cf-nDNA is accessible to IP.

**Figure 1:**
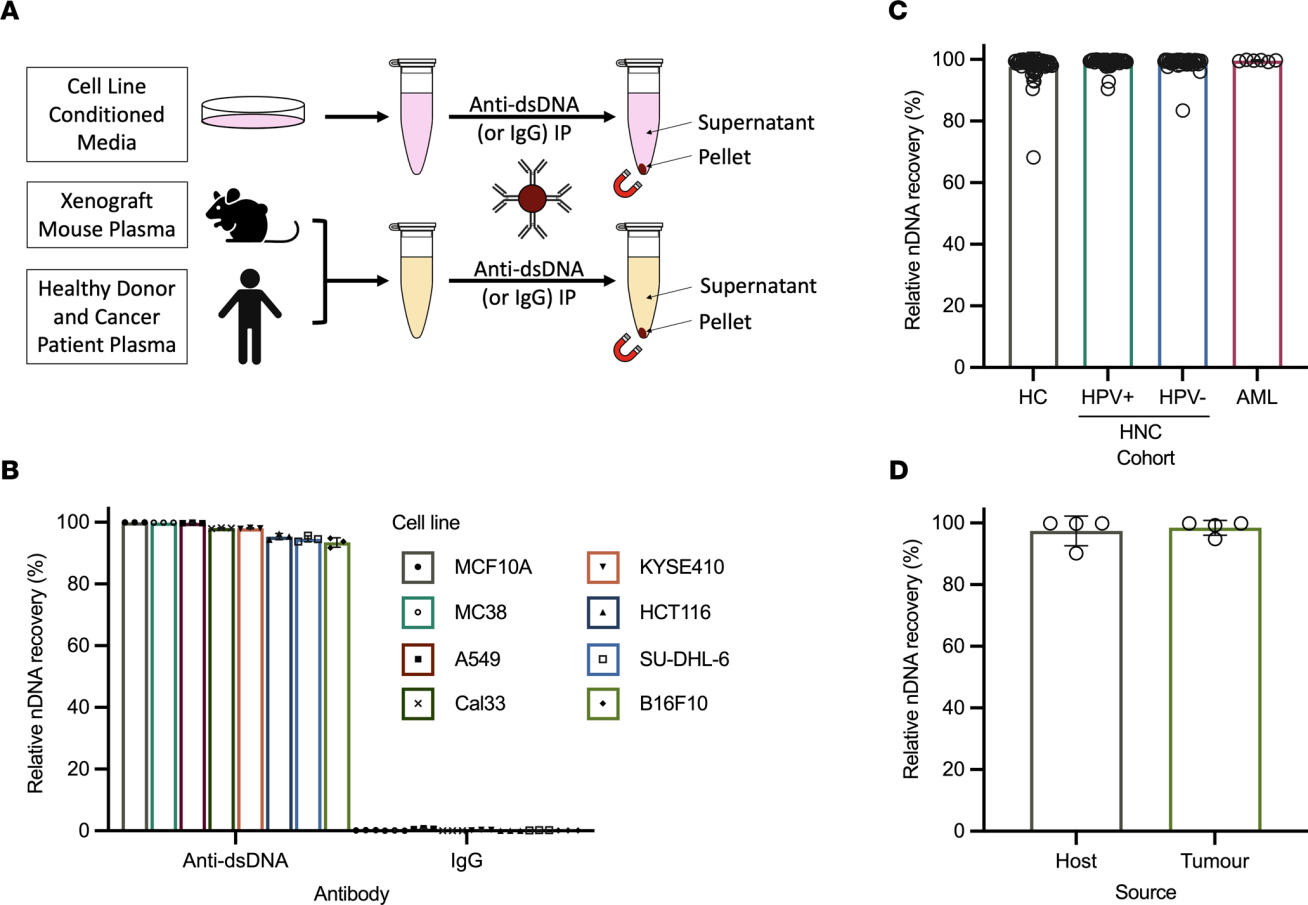
Cell-free nDNA is mostly unprotected from DNA-IP across multiple cancer models. (**A**) Schematic of DNA-IP workflow. Anti-dsDNA antibody was covalently coupled to magnetic beads and added to conditioned media and plasma samples. Exposed DNA was collected in the anti-dsDNA pellet, while inaccessible DNA remained in the supernatant. IgG-bound beads were used as a negative control to evaluate nonspecific DNA binding. (**B**–**D**) Relative recovery of nDNA from conditioned media of cell lines (**B**); HD or HPV^+^ HNC, HPV^–^ HNC, or AML patient plasma (**C**); or Cal33 human oral tongue squamous cell carcinoma xenograft mouse plasma (**D**) by DNA-IP. IgG control IP relative recovery is included for cell line–conditioned media samples (**B**). Human cohorts: HD *n* = 50, HPV^+^ HNC *n* = 49, HPV^–^ HNC *n* = 44, AML *n* = 6; *n* = 4 for mouse cohort.

We established and optimized a DNA-IP strategy along with relevant downstream assays to characterize cfDNA from various biological samples (Figure 1A). We performed DNA-IP on purified genomic DNA and observed high efficiency and relative recovery (calculated by dividing the amount of DNA in the pellet fraction by the sum of DNA in the pellet and supernatant fractions) using the Qubit dsDNA high-sensitivity assay (Invitrogen, Thermo Fisher Scientific, Supplemental Figure 1A and Supplemental Table 1; supplemental material available online with this article; https://doi.org/10.1172/jci.insight.159590DS1). We did not detect any dsDNA in the supernatant fraction, so we next sought to confirm the Qubit results using a more sensitive assay that could detect both single- and double-stranded DNA. We conducted quantitative PCR (qPCR) using primers targeting the repetitive element, long interspersed nuclear element 1 (*LINE1*); the high copy number of *LINE1* in vertebrate genomes allows for ultrasensitive and species-specific nDNA detection (29). *LINE1* qPCR showed the virtually complete relative recovery of purified genomic DNA after DNA-IP, with close to zero detectable nDNA remaining in the supernatant fraction (Supplemental Figure 1A and Supplemental Table 1).

Beginning with conditioned media from a panel of 7 cancer cell lines (A549 [human non–small cell lung carcinoma], Cal33 [human oral tongue squamous cell carcinoma], HCT116 [human colon adenocarcinoma], KYSE410 [human esophageal squamous cell carcinoma], SU-DHL-6 [human large B cell lymphoma], B16F10 [murine melanoma], and MC38 [murine colon adenocarcinoma]) and 1 nontumorigenic cell line (MCF10A [breast epithelium]), we used DNA-IP to capture exposed cfDNA. Across all cell lines, nDNA had relative recoveries >93% (Figure 1B and Supplemental Table 1; range: 93.46%–99.97%). Despite differences in cf-nDNA abundance between cell lines (Supplemental Figure 1B), recovery was not correlated with nDNA concentration (*R*^2^ = 0.1641; *P* = 0.2675). Notably, 5 cell lines showed cf-nDNA recovery > 98% (MCF10A: 99.97% ± 0.001%; MC38: 99.88% ± 0.026%; A549: 99.77% ± 0.089%; Cal33: 98.11% ± 0.151%; and KYSE410: 98.06% ± 0.176%), while the remaining 3 cell lines yielded slightly lower cf-nDNA recovery (HCT116: 95.36% ± 0.984%; SU-DHL-6: 94.65% ± 0.967%; and B16F10: 93.46% ± 1.556%). Moreover, virtually no cf-nDNA was recovered using an IgG control antibody, highlighting the specific DNA-enriching ability and low background of this assay (Figure 1B; range: 0.00–0.01 ng).

We next conducted DNA-IP on plasma samples from healthy human donors (HD), a cohort of patients with HPV-positive head and neck squamous cell carcinoma (HPV^+^ HNC), a cohort of patients with HPV-negative HNC (HPV^–^ HNC), and a cohort of patients with acute myeloid leukemia (AML) carrying the nucleophosmin 1 (*NPM1*) type A mutation at the time of diagnosis. We observed variability in cf-nDNA abundance both within and between cohorts (Supplemental Figure 1C) but saw consistently high relative recovery of cf-nDNA in the pellet (HD: 97.72% ± 4.663%, *n* = 50; HPV^+^ HNC: 99.01% ± 1.662%, *n* = 49; HPV^–^ HNC: 98.87% ± 2.497%, *n* = 44; AML: 99.61% ± 0.239%, *n* = 6) (Figure 1C and Supplemental Table 1). In addition, we analyzed plasma collected from HPV^–^ HNC xenograft mice transplanted with cultured human Cal33 tumor cells. DNA-IP yielded percentage DNA recoveries of 97.43% (±4.844%, *n* = 4) for host (i.e., mouse) cf-nDNA and 98.45% (±4.029%, *n* = 4) for tumor (i.e., human) cf-nDNA (Figure 1D, Supplemental Figure 1D, and Supplemental Table 1).

Taken together, the results from these DNA-IP panels suggest that the vast majority of cf-nDNA in conditioned media and plasma is accessible to DNA-IP and therefore likely not protected within membranous structures.

### Cell-free nDNA is nucleosomal and not EV associated.

While our DNA-IP panel demonstrated high percentages of cf-nDNA recovery, we sought to further explore the potential involvement of EVs in cfDNA structure. We proceeded to use MCF10A, B16F10, and HCT116 for subsequent experiments due to their unique organisms of origin, pathophysiological states, and proportions of cf-nDNA recovery (Figure 1B and Supplemental Figure 1B).

**Figure 2:**
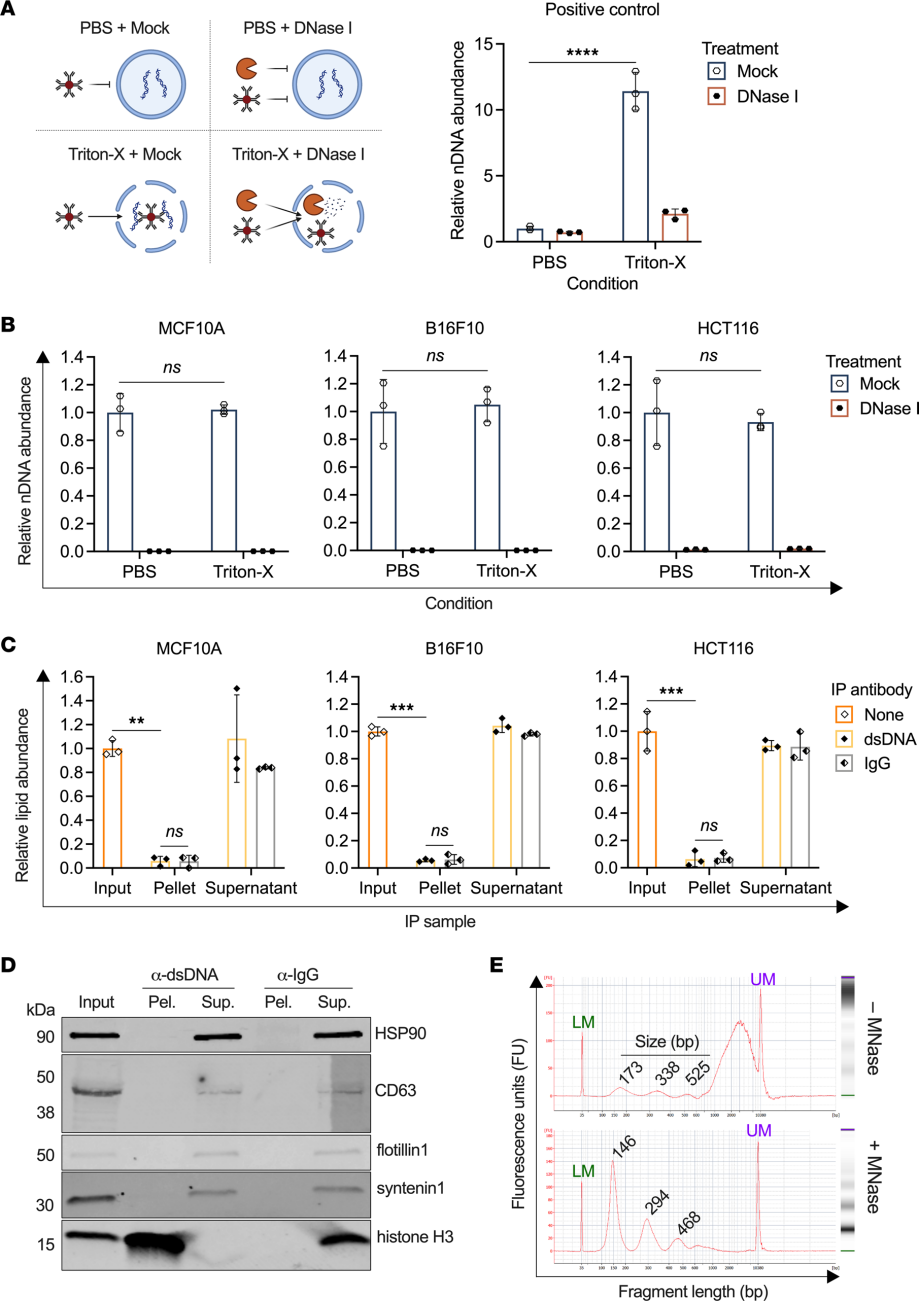
Cell-free nDNA from conditioned media is nucleosomal and not vesicle associated. (**A**) *Left:* Schematic of permeabilization/degradation assay on membrane-protected DNA. Samples were treated with either PBS or Triton X-100 (i.e., the condition) followed by subsequent treatment with either PBS mock or DNase I (i.e., the treatment), before being subjected to DNA-IP. *Right:* HCT116 cells were used as a positive control for membrane-protected DNA subjected to permeabilization/degradation assay. Values were normalized to the PBS/mock treatment. (**B**) Permeabilization/degradation assays on conditioned media from MCF10A, B16F10, and HCT116 cells. Values were normalized to their respective PBS/mock treatment. (**C**) DNA-IP was performed on conditioned media from MCF10A, B16F10, and HCT116 cells, and lipid content in each fraction was quantified using a modified phospho-sulfo-vanillin assay. Values were normalized to their respective input fraction. (**D**) Immunoblotting of common EV markers HSP90, CD63, flotillin1, and syntenin1, as well as histone H3, after DNA-IP of conditioned media. Representative image of blot from HCT116-conditioned media. See complete unedited blots in the supplemental material. (**E**) Cell-free DNA from untreated conditioned media (top) or MNase-treated media (bottom) was purified, and fragment sizes were quantified by BioAnalyzer. Shown are both the BioAnalyzer electropherogram and gel image for each treatment. The green lower marker (LM) is 35 bp, and the purple upper marker (UM) is 10,380 bp. Representative data from HCT116-conditioned media. ns, not significant; **P < 0.01, ***P < 0.001, ****P < 0.0001, ordinary 2-way ANOVA with Tukey’s multiple-comparison test (**A** and **B**); unpaired 2-sided *t* test with Welch’s correction (**C**).

We began by applying a permeabilization/degradation assay to investigate whether cf-nDNA was encapsulated by lipid membrane structures (Figure 2A). Conditioned media samples were pretreated with either PBS as a negative control or the membrane-permeabilizing nonionic detergent Triton X-100, followed by incubation with either PBS mock treatment or DNase I to degrade accessible DNA. Following these treatments, samples were subjected to DNA-IP, and cf-nDNA was quantified by qPCR. Intact HCT116 cells were used as a positive control, as their nDNA is protected by both the plasma membrane and the nuclear envelope. As expected, membrane-protected DNA showed a significant increase in DNA abundance by approximately 11-fold after membrane permeabilization (*P* < 0.0001), demonstrating membrane protection from the DNA-IP antibody in the absence of Triton X-100 (Figure 2A). In contrast, cf-nDNA levels were not significantly different between conditioned media samples treated with PBS and Triton X-100 from all 3 cell lines (MCF10A: *P* = 0.9822; B16F10: *P* = 0.9643; HCT116: *P* = 0.9017), suggesting a lack of cf-nDNA protection within EVs or other membranous structures (Figure 2B). Moreover, DNase I treatment degraded virtually all cf-nDNA in both treatment groups, further reinforcing the accessible nature of this DNA.

We also characterized cf-nDNA topology in EVs isolated by differential ultracentrifugation from conditioned media (Supplemental Figure 2A). The EV pellet did contain small amounts of nDNA, in keeping with previous reports of EV-associated cfDNA (15, 30–33). However, when considering the relative amounts of nDNA in each fraction, we found that >98% remained in the supernatant (Supplemental Figure 2B), suggesting that most cf-nDNA is not EV associated. To determine whether the pellet-contained DNA was membrane protected, we performed DNA-IP on the EV pellet and observed virtually complete recovery of nDNA (Supplemental Figure 2C). Similarly, we did not see a significant increase in nDNA recovery after membrane permeabilization (Supplemental Figure 2D; *P* = 0.6581). These findings from isolated EVs lend further support to our previous conclusion that almost all cf-nDNA is not membrane encapsulated.

While these assays indicated that EVs did not contain substantial quantities of nDNA within their lumina, some studies have reported that EV-associated DNA is primarily bound to the outer EV membrane (34, 35). To test this theory, we performed DNA-IP and quantified lipid content in the resulting pellet and supernatant fractions using an assay optimized for EV detection (36). The DNA-containing pellet fraction had a significantly lower abundance of lipid compared with the conditioned media input (Figure 2C; MCF10A: *P* = 0.0053; B16F10: *P* = 0.0005; HCT116: *P* = 0.0002). Moreover, this fraction did not differ significantly from the IgG control DNA-free pellet fraction (MCF10A: *P* = 0.5211; B16F10: *P* = 0.7303; HCT116: *P* = 0.1728). By comparison, immunoprecipitation of the EV membrane protein CD9 showed a significantly greater abundance of lipid content in the CD9 versus IgG pellet (Supplemental Figure 3A; *P* = 0.0072). These observations implied that cfDNA was not associated in high abundance with the outer membrane of cell-free lipid structures. Subsequent immunoblotting of DNA-IP fractions demonstrated the presence of the common EV markers HSP90, CD63, flotillin1, and syntenin1 in the DNA-IP supernatant rather than the DNA-containing pellet (Figure 2D and Supplemental Figure 3B). In contrast, these markers did coprecipitate with CD9, validating the ability of this approach to identify EV surface components (Supplemental Figure 3C). These results further support the lack of large amounts of DNA associated with outer EV membranes.

Interestingly, DNA-IP with immunoblotting for histone H3 revealed a strong propensity for histone binding by cf-nDNA (Figure 2D and Supplemental Figure 3B). This finding led us to investigate whether cf-nDNA was structured as nucleosomal particles. Fragment size analysis of cfDNA from HCT116-conditioned media revealed small peaks at approximately 173, 338, and 525 bp, roughly corresponding to values expected for mono-, di-, and tri-nucleosomal DNA fragments (Figure 2E). However, these peaks only accounted for 13% of the total cfDNA in this sample; the majority of the cfDNA was found between approximately 800 bp to >10 kb, with a broad peak centered near 3 kb. To investigate whether this large peak comprised cfDNA structured in oligo-nucleosomes, we treated conditioned media with micrococcal nuclease (MNase) to degrade exposed linker DNA between histone core particles. After MNase treatment, this large peak virtually disappeared, and the vast majority of cfDNA (95%) was found in mono-, di-, and tri-nucleosomes (Figure 2E). This finding further demonstrated that larger genomic DNA fragments were accessible to degradation by MNase and, therefore, were not membrane protected. In addition, fragment size distribution of cfDNA captured by DNA-IP was nearly identical to that of cfDNA in the conditioned media input (Supplemental Figure 3D).

Taken together, these findings indicate that cf-nDNA in culture is neither carried within the EV lumen, nor associated with the outer EV membrane in large amounts, but rather exists predominantly in accessible mono- and oligo-nucleosome particles.

### Topologically distinct cfDNA subsets are released via different biogenesis pathways.

**Figure 3:**
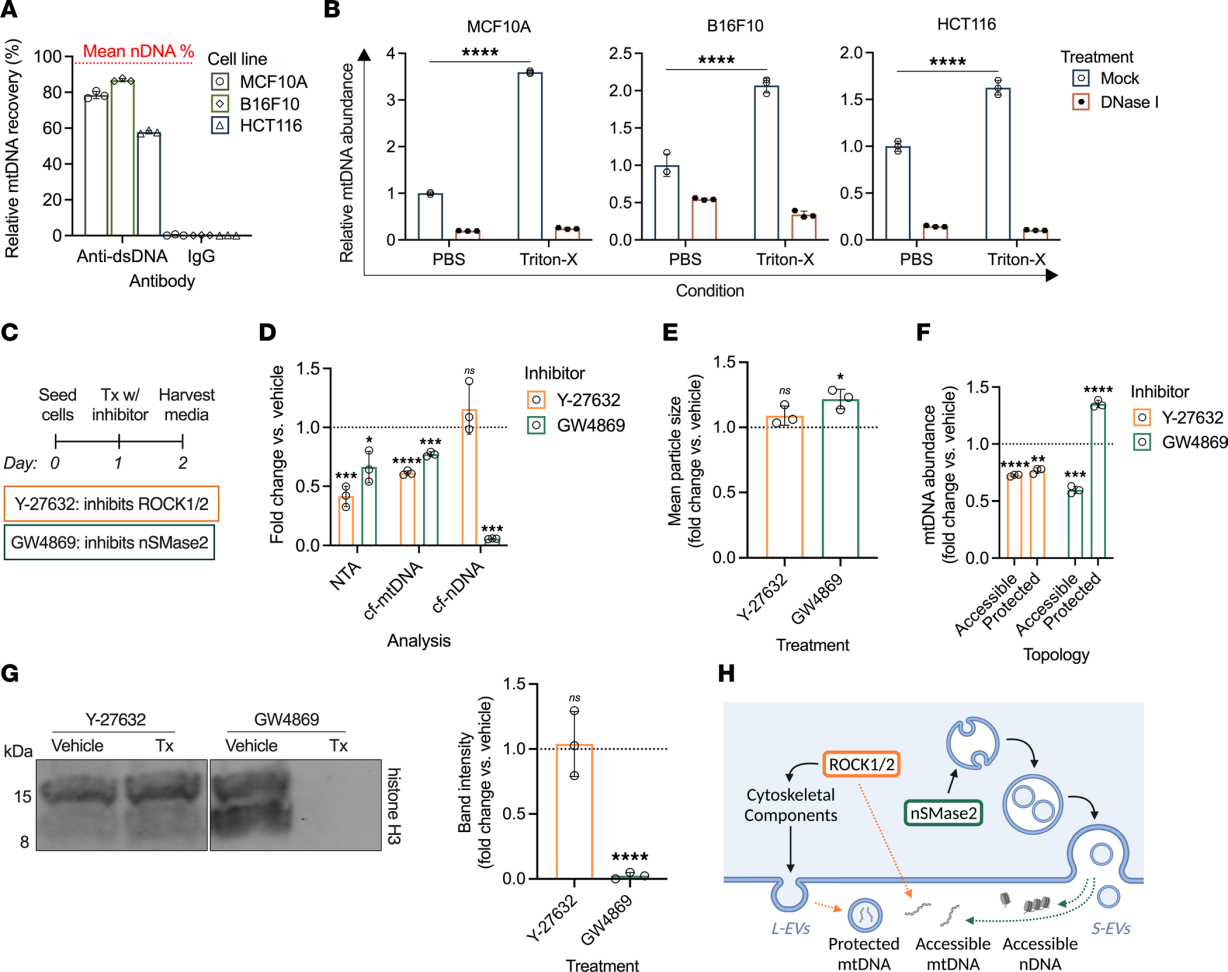
Distinct cellular mechanisms regulate the release of protected mtDNA and accessible mtDNA and nDNA. (**A**) Relative recovery of mtDNA from conditioned media of MCF10A, B16F10, and HCT116 cells by DNA-IP and IgG control. Dotted red line indicates mean relative recovery of nDNA from these cell lines. (**B**) Permeabilization/degradation assays on conditioned media from MCF10A, B16F10, and HCT116 cells. Values were normalized to their respective PBS/mock treatment. (**C**) Timeline of EV inhibitor treatment. HCT116 cells were seeded on day 0, followed by treatment on day 1 with Y-27632 or GW4869; control treatment groups received equivalent volume of vehicle. Media were harvested from each treatment group on day 2 and subjected to downstream analyses. (**D**) *Left:* Particle concentration in conditioned media quantified by nanoparticle tracking analysis (NTA). *Middle:* cf-mtDNA concentration in conditioned media. *Right:* cf-nDNA concentration in conditioned media. Values were normalized to the vehicle control. (**E**) Mean particle size for each inhibitor, as determined by NTA, normalized to the vehicle control. (**F**) Abundance of mtDNA in the pellet (i.e., accessible) or supernatant (i.e., protected) after DNA-IP of inhibitor-treated media, normalized to the vehicle control for each fraction. (**G**) *Left*: Immunoblotting of histone H3 after histone IP of conditioned media from vehicle- and inhibitor-treated cells. Representative image of blot from HCT116-conditioned media. *Right*: Histone H3 band intensity, normalized to the vehicle control. (**H**) Schematic depicting ROCK1/2- and sMNase2-regulated mechanisms of accessible and protected cfDNA. ROCK1/2 mediates biogenesis of large EVs, which contain mtDNA; independently, ROCK1/2 also contributes to the pool of accessible cf-mtDNA. Conversely, sMNase mediates release of accessible nDNA and mtDNA independent of its role in small EV biogenesis. *P < 0.05, **P < 0.01, ***P < 0.001, ****P < 0.0001, ordinary 2-way ANOVA with Tukey’s multiple-comparison test (**B**); unpaired 2-sided *t* test (**D**–**G**).

In addition to nDNA, mtDNA contributes to the overall pool of cfDNA and has been implicated as a potential EV cargo (13). To explore cf-mtDNA structure, we again subjected conditioned media to DNA-IP but this time quantified mtDNA using primers specific to the mitochondrial protein-coding gene NADH-ubiquinone oxidoreductase chain 1 (*MTND1*). Cell-free mtDNA from conditioned media showed markedly lower relative recovery (MCF10A: 78.49% ± 2.019%; B16F10: 86.80% ± 0.928%; HCT116: 57.82% ± 0.878%) compared with corresponding cf-nDNA recovery values (Figure 3A and Supplemental Figure 4). Strikingly, cf-mtDNA abundance from permeabilization/degradation assay samples demonstrated a significant increase in relative DNA abundance after membrane permeabilization versus the untreated control (Figure 3B; MCF10A: +259.69% ± 6.92%, *P* < 0.0001; B16F10: +109.14% ± 24.28%, *P* < 0.0001; HCT116: +62.57% ± 1.41%, *P* < 0.0001). Interestingly, DNase I treatment did not fully degrade cf-mtDNA in either membrane-permeabilized or nonpermeabilized samples (Figure 3B), in contrast to our observations of cf-nDNA (Figure 2B); as the DNase-resistant population of cf-mtDNA was in similar abundance despite EV permeabilization, this finding may reflect an alternative or currently unknown protective mechanism (37).

Based on our cell culture findings showing differential EV association between cf-nDNA and cf-mtDNA, we next investigated whether inhibiting EV biogenesis could modulate cfDNA composition. To accomplish this, we employed 2 inhibitors: Y-27632, a competitive inhibitor of rho-associated protein kinase 1 and 2 (ROCK1/2) that blocks cytoskeleton-mediated EV formation from the plasma membrane, and GW4869, a noncompetitive inhibitor of neutral sphingomyelinase 2 (nSMase2) that interferes with endosomal trafficking and multivesicular body formation (38, 39). HCT116 cells were seeded on day 0 and treated with 1 μM Y-27632 or 10 μM GW4869 on day 1; media were harvested 24 hours following treatment (Figure 3C). Nanoparticle tracking analysis (NTA) demonstrated significant decreases in EV concentration following treatment with either inhibitor compared with the vehicle control (Figure 3D; Y-27632: *P* = 0.0001; GW4869: *P* = 0.0160). Subsequent cf-mtDNA quantification demonstrated significantly lower abundance in inhibitor-treated conditioned media versus the vehicle control (Figure 3D; Y-27632: *P* < 0.0001; GW4869: *P* = 0.0005), indicating potential involvement of EVs in mtDNA release.

We also quantified cf-nDNA and found that while Y-27632 treatment did not alter cf-nDNA abundance, media from GW4869-treated cells contained markedly less cf-nDNA than the control (Figure 3D; Y-27632: *P* = 0.3163; GW4869: *P* = 0.0001). This observation was surprising given our earlier finding that cf-nDNA was overwhelmingly not EV-associated. Interestingly, there may be considerable overlap in the pathways involved in the release of nucleosomes and EVs (12, 13). Therefore, we next sought to determine whether our observed changes in cfDNA abundance after inhibitor treatment reflected EV-protected DNA or accessible nucleosomal particles. We investigated trends in particle size as determined by NTA and found that while mean particle size in Y-27632 media did not significantly differ from the vehicle control (*P* = 0.1317), the mean particle size in GW4869 media was significantly larger (Figure 3E; *P* = 0.0207). The frequency distribution of particles in GW4869 media showed that this increase in mean particle size was driven by a decrease in small sub-EV particles below ~40 nm and an increase in large-EV-sized particles above ~200 nm (Supplemental Figure 5A, left). Conversely, the Y-27632 particle frequency distribution suggested only a small decrease in particles below ~40 nm and variability in the abundance of larger particles (Supplemental Figure 5A, right).

As such, we hypothesized that these inhibitors were influencing the release of small DNA-containing particles in addition to EVs. To investigate this, we probed changes in mtDNA topology in response to ROCK1/2 or nSMase2 inhibition by conducting DNA-IP on media from vehicle- and inhibitor-treated cells and quantifying mtDNA in each fraction. Interestingly, both accessible (*P* < 0.0001) and protected (*P* = 0.0012) cf-mtDNA decreased after Y-27632 treatment, implicating ROCK1/2-dependent pathways in the release of EV- and non–EV-associated mtDNA (Figure 3F). Strikingly, GW4869 treatment resulted in a relative decrease in accessible mtDNA (*P* = 0.0002) and a relative increase in protected mtDNA (*P* < 0.0001), suggesting that nSMase2 is involved in non–EV-associated mtDNA release but not EV-associated mtDNA release (Figure 3F).

Based on the decrease in cf-nDNA after GW4869 treatment, we hypothesized that the sMNase pathway was responsible for the release of accessible nucleosomal particles. Remarkably, treatment with GW4869 but not Y-27632 resulted in near-total depletion of cell-free histones (Figure 3G; Y-27632: *P* = 0.8003; GW4869: *P* < 0.0001). Together with our previous evidence that cf-nDNA is histone bound (Figure 2, D and E), this finding indicates that the release of nonmembranous nucleosomal particles is regulated by nSMase2. Although nSMase2 has also been reported to regulate ceramide-mediated apoptosis (39), cell viability (Supplemental Figure 5B; *P* = 0.5257) and caspase activity (Supplemental Figure 5C; *P* = 0.0929) were not significantly reduced by GW4869 treatment. By comparison, direct caspase inhibition with Z-VAD-FMK yielded markedly lower normalized caspase activity (Supplemental Figure 5C; *P* = 0.0131). These results indicated that the decrease in cf-nDNA abundance following nSMase2 inhibition was independent of apoptotic pathways.

Taken together, these findings indicate that ROCK1/2 regulates release of 2 topologically distinct mtDNA populations, while active release of nucleosomal nDNA and a subset of accessible mtDNA is mediated by nSMase2 (Figure 3H).

### Plasma-derived cf-nDNA is nonvesicular.

Unlike conditioned media, in which cultured cells are the lone source of cfDNA, plasma contains cfDNA from multiple sources. In fact, a large portion of plasma cfDNA derives from hematopoietic cells, even in tumor-bearing patients (8, 9). Therefore, we first sought to determine the degree to which total cf-nDNA is protected within membranous structures in human plasma. As previously mentioned, DNA-IP yielded near-total relative recovery of cf-nDNA in both healthy donor and cancer patient plasma samples (Figure 1C).

**Figure 4:**
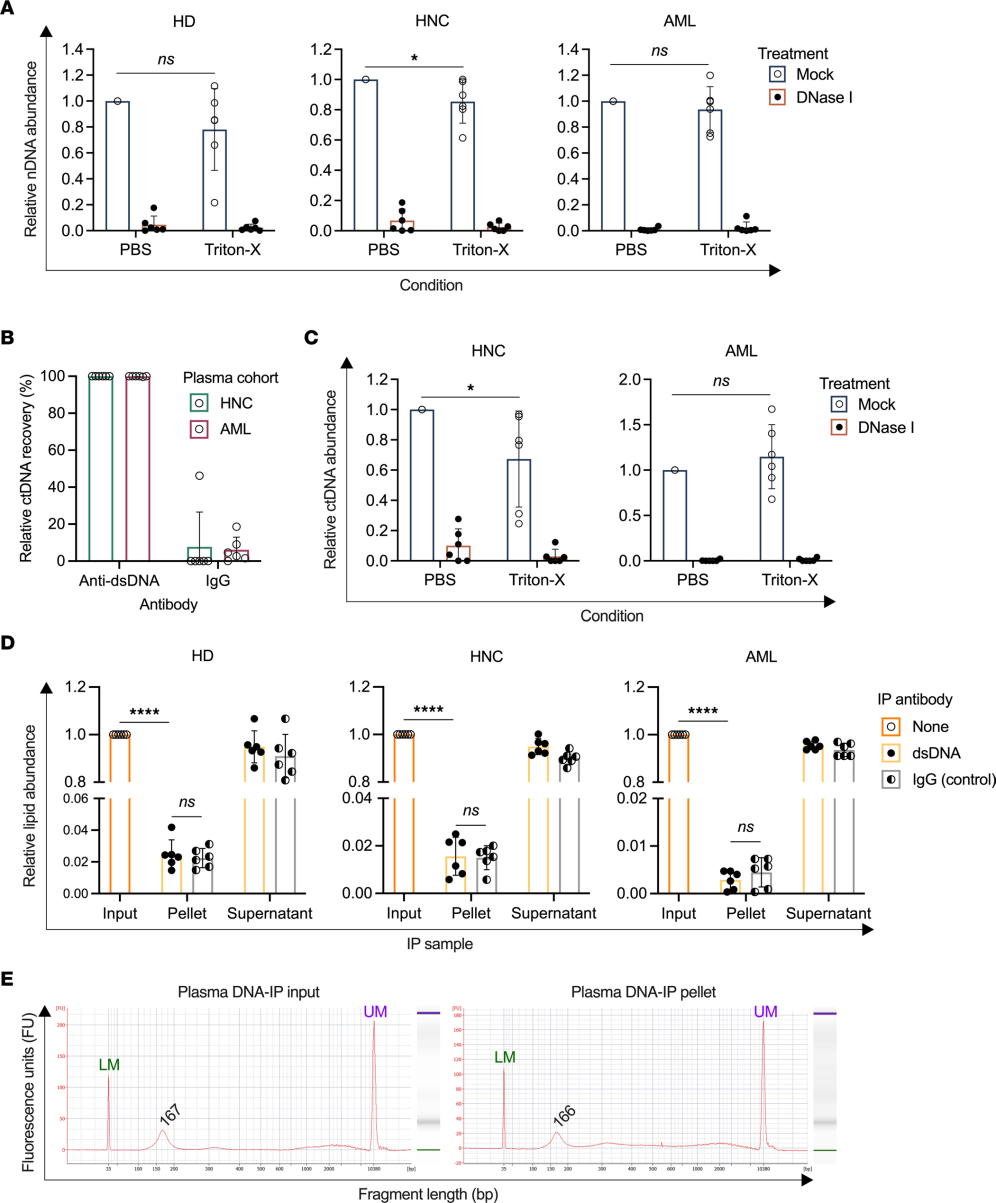
Plasma-derived cf-nDNA is not vesicle associated in a healthy or tumor-bearing state. (**A**) Permeabilization/degradation assays on select samples of HD (left), HPV^+^ HNC patient (middle), and AML patient (right) plasma. Values were normalized to their respective PBS/mock treatment. (**B**) Relative recovery of ctDNA from select samples of HPV+ HNC and AML patient plasma. (**C**) ctDNA abundance after permeabilization/degradation assay on select samples of HPV^+^ HNC and AML patient plasma. Values were normalized to the PBS/mock treatment. (**D**) DNA-IP was performed on select samples of HD (left), HPV^+^ HNC patient (middle), and AML patient (right) plasma, and lipid content in each fraction was quantified using a modified phospho-sulfo-vanillin assay. Values were normalized to their respective input fraction. (**E**) Human plasma was subjected to DNA-IP, and DNA fragment sizes in the input and DNA-IP pellet fractions were quantified by BioAnalyzer. Shown are both the BioAnalyzer electropherogram and gel image for each fraction. The green lower marker (LM) is 35 bp, and the purple upper marker (UM) is 10,380 bp. Representative data from 1 AML patient plasma sample. *P < 0.05, ****P < 0.0001; ordinary 2-way ANOVA with Tukey’s multiple-comparison test (**A** and **C**); unpaired 2-sided *t* test with Welch’s correction (**D**). *n* = 6 for human cohorts; *n* = 4 for mouse cohort.

To further probe the apparent unprotected nature of cf-nDNA, plasma was subjected to permeabilization/degradation assays. The relative abundance of nDNA after DNA-IP was not significantly elevated in detergent-treated samples from either healthy donors or cancer patients (Figure 4A). Next, we specifically quantified the tumor-derived fraction of cf-nDNA, termed circulating tumor DNA (ctDNA), using droplet digital PCR (ddPCR) targeting HPV16 E6 and E7 sequences and the *NPM1* mutant type A sequence prevalent in the HPV^+^ HNC and AML patient cohorts, respectively (Supplemental Figure 6, A and B). Consistent with our earlier findings, we observed virtually complete relative recovery of ctDNA by DNA-IP (Figure 4B) and no significant increase in ctDNA abundance between untreated and detergent-treated samples (Figure 4C).

To further validate the apparent absence of both non–tumor-derived and tumor-derived cf-nDNA in plasma EVs, we analyzed plasma collected from mice xenografted with cultured human Cal33 tumor cells. As described above, DNA-IP yielded high relative recoveries for both host and tumor-derived cf-nDNA (Figure 1D). Furthermore, we found no significant difference between untreated and detergent-treated permeabilization/degradation assay samples for either source of cf-nDNA (Supplemental Figure 6C; host: *P* = 0.6208; tumor: *P* = 0.7726).

As these analyses suggested that both non–tumor- and tumor-derived plasma cf-nDNA was not encapsulated within membrane-bound structures, we further explored the structure and topology of this DNA. DNA-IP with lipid quantification showed no significant difference in lipid abundance between the anti-dsDNA and IgG pellets in any plasma sample cohort (Figure 4D; HD: *P* = 0.6204; HPV^+^ HNC: *P* = 0.8817; AML: *P* = 0.3186). Next, we investigated whether cf-nDNA structure in plasma reflected the nucleosomal structure observed in conditioned media. Fragment size analysis yielded a single mode at 167 bp in the input fraction and 166 bp in the DNA-IP pellet, corresponding to the expected length of mono-nucleosome particles in plasma (40) (Figure 4E). We also probed plasma-derived cf-nDNA structure by determining its DNA integrity index (DII). This surrogate for DNA fragment size was obtained by comparing the qPCR readouts of each sample using 2 nested primer sets that generate a relatively short (82 bp) and long (224 bp) amplicon from the same locus (see Methods). While we observed slight variations in DII between individual patients within each cohort, DII did not differ significantly between input DNA and DNA-IP pellet DNA (Supplemental Figure 6D; HD: *P* > 0.9999; HPV^+^ HNC: *P* = 0.2751; AML: *P* = 0.9921). These results support that DNA fragment size is conserved by DNA-IP in plasma, thereby suggesting that neither short nor long cf-nDNA fragments exist in substantial quantities in membrane-protected forms.

Together, these results indicate that plasma-derived cf-nDNA is not carried abundantly in vesicular structures and instead circulates as accessible nucleosomal fragments.

### A subset of cf-mtDNA is membrane protected in plasma.

**Figure 5:**
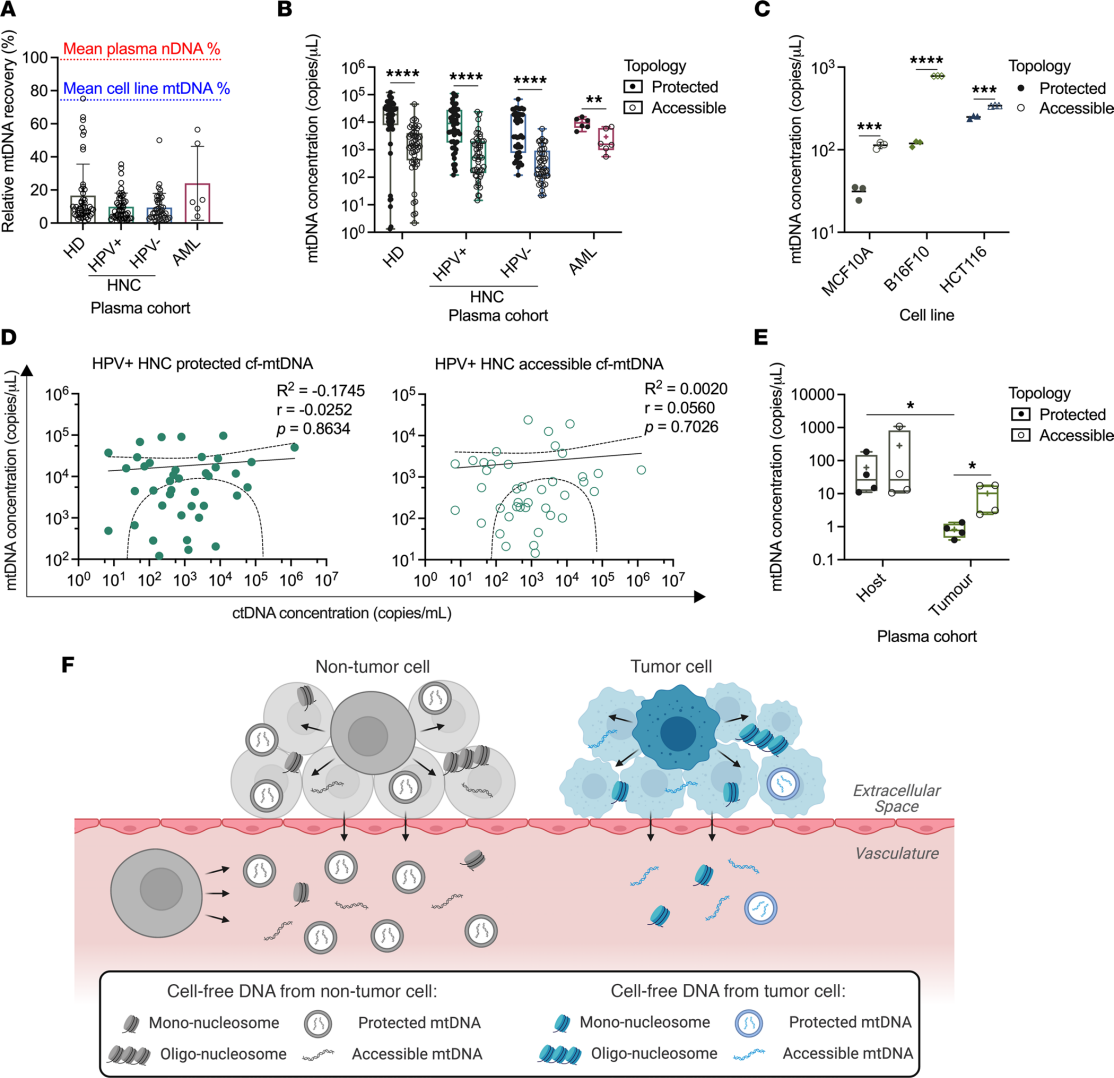
Cell-free mtDNA in tumor-bearing state derives predominantly from nontumor cells. (**A**) Relative recovery of mtDNA from HD and HPV^+^ HNC, HPV^–^ HNC, and AML cancer patient plasma by DNA-IP. Dotted red line indicates the mean relative recovery of plasma nDNA. Dotted blue line indicates the mean relative recovery of mtDNA from cell line–conditioned media. (**B** and **E**) Concentration of protected and accessible cf-mtDNA in HD and patient plasma (**B**) or Cal33 xenograft plasma (**E**). Data are represented as box-and-whiskers plots displaying mean (+ symbol), median (horizontal line), minimum (lower whisker), 25th percentile (lower bound of box), 75th percentile (upper bound of box), and maximum (upper whisker). (**C**) Concentration of protected and accessible cf-mtDNA in cell line–conditioned media. (**D**) Plots of protected (left) and accessible (right) cf-mtDNA in HPV^+^ HNC patient plasma versus tumor burden (as measured by ctDNA). Data are fit with a nonlinear log-log regression and dashed lines represent 95% confidence interval. (**F**) An updated schematic of cfDNA origins and structure in tumor-bearing individuals reflecting our findings. Cell-free nDNA from both nontumor and tumor cells is not associated with EVs and exists as mono- and oligo-nucleosome particles. Conversely, a portion of cf-mtDNA from both tumor and nontumor cells is protected within membranous structures. However, cf-mtDNA from nontumor cells is more abundant, and a greater proportion is membrane protected than cf-mtDNA from tumor cells. *P < 0.05, **P < 0.01, ***P < 0.001, ****P < 0.0001, nonparametric 2-sided *t* test with Mann-Whitney test (**B** and **E**); unpaired 2-sided *t* test (**C**); nonlinear log-log regression with Spearman correlation (**D**). Human cohorts: HD *n* = 50, HPV^+^ HNC *n* = 49, HPV^–^ HNC *n* = 44, AML *n* = 6; *n* = 4 for mouse cohort.

We next investigated cf-mtDNA topology in plasma. Strikingly, there was markedly lower relative recovery of plasma cf-mtDNA compared with both plasma cf-nDNA and cf-mtDNA from cancer cell lines (Figure 5A). Relative recovery of cf-mtDNA by DNA-IP averaged 16.65% (±18.98%, *n* = 50) for HD plasma, 9.88% (±8.26%, *n* = 49) for HPV^+^ HNC plasma, 9.34% (±8.66%, *n* = 44) for HPV^–^ HNC plasma, and 24.02% (±22.32%, *n* = 6) for AML plasma (Figure 5A).

Aside from this difference in accessibility between plasma cf-mtDNA and cell line–conditioned media cf-mtDNA, other attributes appeared largely consistent between the 2 sources. Permeabilization/degradation assays on select samples showed significant increases in relative mtDNA abundance after permeabilization in HD and cancer patient plasma (Supplemental Figure 7A). Plasma-derived cf-mtDNA was also somewhat resistant to DNase I degradation both with and without membrane permeabilization, consistent with cell line data.

Based on our observation that cf-mtDNA accessibility in plasma was much lower than it was in conditioned media from cancer cell lines, we hypothesized that membrane-protected cf-mtDNA in plasma derived mostly from nontumor cells. Interestingly, the mean abundance of protected cf-mtDNA was similar among the HD and cancer patient cohorts (Figure 5B). Within each cohort, protected cf-mtDNA in plasma was significantly more abundant than accessible cf-mtDNA (Figure 5B; HD: *P* < 0.0001; HPV^+^ HNC: *P* < 0.0001; HPV^–^ HNC: *P* < 0.0001; AML: *P* = 0.0087), in contrast to cf-mtDNA from cell line–conditioned media (Figure 5C; MCF10A: *P* = 0.0003; B16F10: *P* < 0.0001; HCT116: *P* = 0.0002). We also observed no correlation between membrane-protected cf-mtDNA (Figure 5D, left; *P* = 0.8634), accessible cf-mtDNA (Figure 5D, right; *P* = 0.7026), or relative cf-mtDNA recovery (Supplemental Figure 7B) and ctDNA concentration — a surrogate of tumor burden — indicating that protected cf-mtDNA release is independent of tumor burden and therefore likely derives largely from nontumor cells. Similarly, neither cf-mtDNA subset was significantly correlated with ctDNA (i.e., *NPM1* variant allele frequency within cfDNA) of patients with AML (Supplemental Figure 7C).

To further investigate the origin of cf-mtDNA in tumor-bearing individuals, we again used plasma from mice xenografted with human Cal33 cells. We found that protected cf-mtDNA from host cells was significantly more abundant than that derived from tumor cells (Figure 5E; *P* = 0.0286). Moreover, significantly more cf-mtDNA derived from tumor cells was accessible than protected as determined by DNA-IP (Figure 5E; *P* = 0.0286) and permeabilization/degradation assays (Supplemental Figure 7D). Interestingly, we did not observe lower accessible versus protected cf-mtDNA from host cells, as seen in human cohort plasma. This apparent difference may be attributed to incongruency between our mouse and human models; for example, host cells in these mice are deficient for many leukocyte subsets known to contribute to the pool of host-derived cfDNA (3), and the proportion of ctDNA in xenograft mouse plasma (63.52% ± 32.68%; Supplemental Figure 1D) was much higher than would be expected in human plasma (41, 42). Nonetheless, these results corroborate our observations from human plasma and cell line–conditioned media and indicate that cf-mtDNA from nontumor cells, a greater proportion of which is membrane protected, is the main contributor to the pool of cf-mtDNA in tumor-bearing individuals.

Overall, our results from preclinical models and clinical samples support a revised model of cfDNA structure and distribution according to its subcellular and cellular origins in the tumor-bearing state (Figure 5F). Cell-free nDNA exists predominantly as non–EV-associated nucleosomal particles, and distinct subsets of cf-mtDNA are either accessible or protected within membranous structures. Furthermore, cf-mtDNA released from tumor cells is mostly accessible. Finally, nontumor cells account for the majority of cf-mtDNA in tumor-bearing individuals, and a greater proportion of this cf-mtDNA is protected.

### Accessible cf-mtDNA may serve as a novel cancer biomarker.

**Figure 6:**
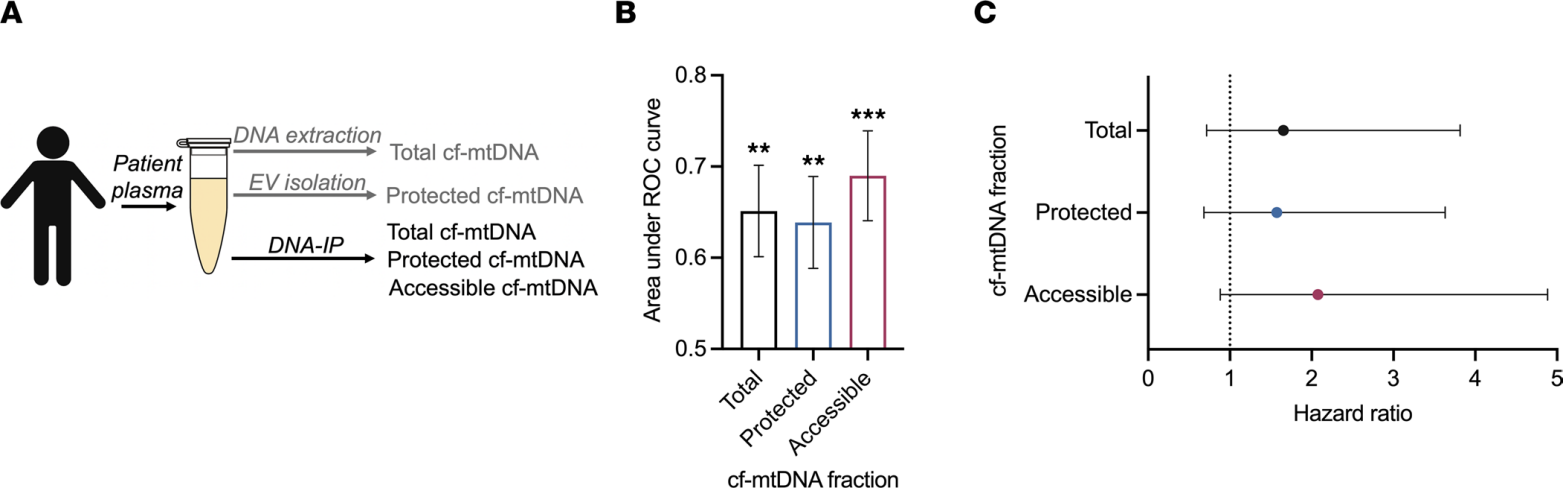
Accessible cf-mtDNA is a potentially useful cancer biomarker. (**A**) Schematic outlining the implementation of DNA-IP as a clinical tool to distinguish between multiple topological subsets of cf-mtDNA. (**B**) Area under the receiver operating characteristic curve (AUROC) for cf-mtDNA subsets in patients with HNC. Data are reported as AUROC ± SEM. (**C**) Survival analysis of patients with HNC according to abundance of total, protected, and accessible cf-mtDNA abundance, stratified into low or high based on median abundance of the specified mtDNA subset. Recurrence-free survival between above-median and below-median patients are compared using the log-rank test, and data are reported as a forest plot with error bars showing 95% confidence interval. *P < 0.01, ***P < 0.001, ROC analysis (**B**); log-rank test (**C**). *n* = 93 for HNC cohort.

Cell-free mtDNA has emerged as a novel liquid biopsy substrate in cancer; however, studies to date have relied on total cf-mtDNA and/or protected cf-mtDNA as liquid biopsy analytes (43, 44). Having established that cf-mtDNA from cancer cells is predominantly accessible, we next sought to explore the potential clinical utility of DNA-IP to enrich for this subset of interest (Figure 6A). We analyzed plasma from patients with HNC (*n* = 93 total; 49 HPV^+^, 44 HPV^–^) used in earlier experiments; demographic and clinical information are reported in Supplemental Table 2. We first investigated the diagnostic capability of accessible cf-mtDNA compared with total and protected cf-mtDNA. Interestingly, the area under the receiver operating characteristic curve (AUROC) was 0.6899 for accessible cf-mtDNA versus 0.6512 for total cf-mtDNA and 0.6387 for protected cf-mtDNA in patients with HNC (Figure 6B; total: *P* = 0.0029; protected: *P* = 0.0063; accessible: *P* = 0.0002). These findings demonstrated the ability of accessible cf-mtDNA to distinguish patients with cancer from healthy controls more accurately at the time of diagnosis. This observation likely reflects our earlier observation that tumor cells released proportionally more accessible than protected cf-mtDNA (Figure 5).

In addition, we examined the potential prognostic value of measuring accessible cf-mtDNA in these patients. We conducted survival analysis by stratifying patients into those with either high (i.e., above median) or low (i.e., below median) total, protected, or accessible cf-mtDNA, based on the median values within each cohort (i.e., HPV^+^ or HPV^–^ HNC). Stratification by accessible cf-mtDNA yielded a greater HR and trended more toward significance compared with total and protected cf-mtDNA (Figure 6C; total: *P* = 0.2411, HR = 1.653 [0.714–3.816]; protected: *P* = 0.2913, HR = 1.574 [0.682–3.633]; accessible: *P* = 0.1066, HR = 2.076 [0.882–4.884]). If validated in larger cohorts, this observation could reflect an association of accessible cf-mtDNA abundance with poor prognosis in HNC patients and — together with our earlier results — illustrates the potential clinical applications of accessible cf-mtDNA enriched by DNA-IP as a cancer biomarker.

## Discussion

Cell-free DNA is being widely promoted as a promising cancer biomarker that informs diagnosis, prognosis, and treatment (8, 9). Similarly, EVs have emerged as a potentially useful reservoir of macromolecules with liquid biopsy applications (45). In addition to their roles as putative biomarkers, cfDNA and EVs have been observed to mediate functional and pathophysiological effects within the tumor microenvironment and at distant sites. As such, the fields of cfDNA and EVs have intersected to stimulate intense study of EV-associated DNA, focusing on its clinical and functional applications (18). However, the structural and biophysical mechanisms by which EVs and cfDNA may or may not be associated have been largely overlooked.

Here, we comprehensively characterized cfDNA in multiple cancer models and observed distinct structural conformations depending on its subcellular and cellular origins. First, we employed a DNA-IP approach on conditioned media to capture exposed cfDNA. Importantly, we found near-complete nDNA recovery across a panel of nontumor and cancer cell lines, suggesting that most cf-nDNA was not protected from antibody binding by enclosure within a membranous vesicle. Strikingly, cf-mtDNA was recovered at markedly lower levels compared with cf-nDNA, indicating that a portion of this DNA was not exposed to IP and indeed was encapsulated in vesicles. Next, we designed a permeabilization/degradation assay to further probe the protected nature of cfDNA. Our results strongly indicate that cf-nDNA was rarely contained within EVs. Furthermore, we used lipid quantification and immunoblotting of EV markers to show that DNA was not bound in large quantities to the outer membrane of EVs. Instead, we observed that cf-nDNA was present in histone-bound structures that demonstrated mono- and oligo-nucleosomal fragment sizes. Our results also revealed that a subset of cf-mtDNA was protected within membranous structures. Moreover, modulation of intracellular trafficking pathways identified distinct mechanisms regulating the release of both accessible and protected cfDNA. Importantly, our cell culture findings were confirmed by similar analyses of HD and cancer patient plasma, as well as mouse xenograft plasma. However, we rather surprisingly observed that within tumor-bearing individuals, the overall abundance and proportion of membrane protection were greater for cf-mtDNA derived from nontumor versus tumor cells. Consequently, we found that cf-mtDNA from tumor cells was predominantly accessible. Based on these findings, we demonstrated that accessible cf-mtDNA enriched by DNA-IP was better suited as a potential diagnostic and prognostic biomarker in cancer compared with total or protected cf-mtDNA.

Until recently, nDNA was widely accepted as a bona fide EV cargo; several studies posited the existence of EV-encapsulated nDNA by quantifying genomic mutations in EV isolates (30, 46, 47). However, in a seminal study on EV composition, Jeppesen et al. found that nDNA was actively released by cancer cells through EV-independent mechanisms (12). Our results align with those of Jeppesen et al. and other recent studies that have further called into question the existence of nDNA within EVs (27, 28). Moreover, we identified nSMase2 as a potential regulator of EV-independent nDNA release.

Conflicting findings on EV contents may stem from incomplete characterization of cfDNA topology in prior studies. For example, EV preparations containing bulk DNA following nuclease treatment (48, 49) may reflect the presence of cf-mtDNA as opposed to cf-nDNA. Similarly, high-resolution imaging approaches that detect EV-associated DNA using DNA-specific dyes or antibodies (31–33, 50) do not distinguish cf-mtDNA from cf-nDNA. To combat these issues, we designed a multicomponent permeabilization/degradation assay with targeted DNA quantification that allowed for discrimination of both nDNA and mtDNA within and outside of membranous structures. Moreover, recent large-scale proteomic studies suggest that DNA-binding proteins are not associated with EVs (51–53). Surface-associated DNA may instead be an artifact of EV isolation, existing as a contaminant in isolated EV samples (54). By applying our DNA-IP assays to raw samples rather than isolated EVs, we demonstrated that DNA does not coprecipitate with common EV surface markers or lipid structures in general.

While nDNA was largely absent from EVs, we observed a subset of membrane-protected mtDNA. This finding corroborates other investigations showing both mtDNA and other mitochondrial components (i.e., mtDNA binding proteins) in EVs (27, 55, 56). However, the mechanisms of mtDNA loading and release by EVs remain unclear. Our EV inhibition assay revealed decreases in both EV-bound and accessible cf-mtDNA after treatment with Y-27632, implicating plasma membrane–derived EVs as carriers of mtDNA with concurrent release of non-EV mtDNA. Future work should aim to further elucidate the subcellular mechanisms of mtDNA packaging and release in EVs and as accessible particles.

In addition to its subcellular origins, we also observed differences in the cellular origin of cf-mtDNA in tumor-bearing individuals. While cf-mtDNA from nontumor cells in this setting was more abundant and membrane-protected than cf-mtDNA from tumor cells, identifying the specific cell types involved, as well as identifying the mechanisms by which the tumor-bearing state mediates mtDNA release from nontumor cells, require further investigation. Notably, the mice used in these experiments were lymphocyte deficient, so other cell types would have been responsible for nontumor cf-mtDNA release.

EV-associated DNA has been suggested as a potential tool in liquid biopsy (22, 24, 57). Our observations that tumor cells release proportionally more accessible than protected cf-mtDNA, and that EVs contain substantial quantities of mtDNA primarily derived from nontumor cells — but little to no nDNA — will help direct biomarker studies. Although cf-mtDNA abundance, mutational status, and fragment size have been proposed as cancer biomarkers (58–60), EV-derived mtDNA has not been widely studied but may harbor additional novel information and/or clinical associations in cancer (43, 61) and other conditions in which cf-mtDNA is prevalent (13). Furthermore, we highlight accessible cf-mtDNA in isolation as an additional — and potentially more informative — liquid biopsy substrate in cancer. Our analyses of cf-mtDNA topology provide a framework for larger scale studies in diverse cohorts.

In addition to its role in liquid biopsy, EV-associated DNA has been implicated in mediating functional processes. For example, Diamond et al. claim that irradiated exosomes deliver immunogenic nDNA to recipient DCs, wherein it mediates an immune response (62). Another report proposes EVs as a delivery vehicle for tumor-derived nDNA, which enters the nucleus of recipient cells and induces upregulation of protumorigenic genes (31). While our findings do not contradict the functional roles of tumor-derived cf-nDNA in these studies, they suggest that very small amounts of EV-associated nDNA may be responsible for these effects or that it may be transferred to recipient cells as nonmembranous nucleosomal particles. Several studies have also found EVs to be involved in mtDNA transport to recipient cells (56, 63, 64). It remains unclear whether membrane-protected or accessible cf-mtDNA mediates intercellular communication, as we found only a subset of mtDNA sequestered within EVs and it was predominantly derived from nontumor cells. Therefore, future work should focus on identifying the mechanisms by which cfDNA is transported to and taken up by the recipient cells in which it functions.

The current study has several limitations. First, our findings are applicable only within the context of cancer patients and healthy individuals. While we strove to use diverse cell lines reflecting different pathologies and organisms, some cancer cells not tested may release cfDNA with unique structural and biophysical properties. In addition, our method of nDNA quantification reported only cfDNA fragments containing the short *LINE1* sequence. Therefore, short repeat sequences in nDNA such as telomeres cannot be excluded as potential EV cargos (65). Similarly, our DNA-IP was specific for dsDNA but not ssDNA; as such, ssDNA remaining in the DNA-IP supernatant may have been amplified by PCR and have unintentionally contributed to the protected proportion of DNA regardless of its actual structure. Furthermore, the markers we used for immunoblotting do not entirely reflect the complex heterogeneity of diverse EV subpopulations. We selected markers in accordance with the most recent Minimal Information for Studies of Extracellular Vesicles guidelines (19), along with new evidence that has since provided an updated set of proposed pan-EV markers (53). In addition, immunoblotting and lipid quantification may not be sensitive enough to detect very small amounts of surface DNA. Moreover, our EV inhibition assays only investigated 2 pathways of EV biogenesis; further analyses should probe other EV trafficking and release mechanisms as they relate to DNA release. Finally, our cancer patient cohorts included 3 types of cancer; future work will determine whether our observations remain consistent across additional cancer types.

In conclusion, we employed a DNA-targeted approach to characterize cfDNA topology in multiple cancer models and human cohorts. We found that cf-nDNA was predominantly nucleosomal and not associated in large quantities with EVs, while a portion of cf-mtDNA was membrane protected but, surprisingly, was derived largely from nontumor cells. We also found that tumor cells released more accessible than protected cf-mtDNA, and enrichment of the accessible fraction by DNA-IP can provide useful diagnostic and prognostic information. Our study supports nucleosomal cf-nDNA as the most compelling liquid biopsy substrate and justifies the discrimination between EV-protected and accessible mtDNA subsets to gain further clinical insights. This study clarifies cfDNA structure and provides promising directions for basic, translational, and clinical investigations.

## Methods

### Cell culture and conditioned media collection.

MCF10A and Cal33 were provided by Fei-Fei Liu at the Princess Margaret Cancer Centre (PMCC). A549 was from Bradley Wouters (PMCC), MC38 from Tracy McGaha (PMCC), and SU-DHL-6 from Robert Kridel (PMCC). B16F10 was from Rama Khokha (PMCC). HCT116 and KYSE410 were purchased from the American Type Culture Collection (ATCC) and MilliporeSigma, respectively. FBS and horse serum used in cell culture media were ultracentrifuged using a Beckman Coulter Optima XPN-80 ultracentrifuge at 120,000*g* for 18 hours at 4°C to remove contaminating EVs. MCF10A cells were grown in phenol-free DMEM/F12 (Wisent Bioproducts) supplemented with horse serum (5%), EGF (20 ng/mL), hydrocortisone (0.5 mg/mL), cholera toxin (100 ng/mL), insulin (10 μg/mL), and 100× penicillin-streptomycin (10 mL/L). HCT116, KYSE410, and SU-DHL-6 cells were grown in phenol-free RPMI-1640 (Wisent Bioproducts) with 10% FBS and 100× penicillin-streptomycin (10 mL/L). B16F10, A549, and Cal33 cells were grown in phenol-free DMEM (Wisent Bioproducts) supplemented with 10% FBS and 100× penicillin-streptomycin (10 mL/L). MC38 cells were grown in phenol-free HyClone McCoy’s 5A media (Cytiva) supplemented with l-glutamine (1.5 mM), sodium bicarbonate (2.2 g/L), 10% FBS, and 100× penicillin-streptomycin (10 mL/L). All cells were grown in air containing 5% CO_2_ at 37°C. To obtain conditioned media, low-passage cells were seeded and incubated undisturbed for 48 hours. Afterward, conditioned media were collected and spun using an Eppendorf 5810R centrifuge at 301*g* for 10 minutes at 4°C to remove cells. Conditioned media were subsequently aliquoted and stored at –20°C.

For immunoblotting experiments, cells were switched to serum-free media prior to 48 hours’ incubation to eliminate contamination with serum-derived albumin. The resulting conditioned media were collected and concentrated with Amicon Ultra-2 Centrifugal Filter Devices with a 3 kDa MW cut-off (MilliporeSigma) using an Eppendorf 5810R centrifuge at 3,180*g* for 70 minutes at room temperature, followed by a recovery spin at 1,000*g* for 2 minutes at room temperature, to increase protein concentration. Concentrated conditioned media samples were pooled, aliquoted, and stored alongside nonconcentrated conditioned media samples at –20°C until use.

### Collection of human plasma samples.

HD samples were obtained from healthy volunteers at PMCC. HNC patient samples were obtained from the PMCC HNC Translational Research program. AML samples were obtained from the Leukemia Tissue Bank at PMCC. Human whole blood was collected into EDTA tubes from healthy donors and from patients with HNC at diagnosis. Plasma was separated from the cell pellet within 2 hours of collection by centrifugation at 2,500*g* for 10 minutes at 4°C followed by aliquoting of plasma and storage at −80°C until use.

### Collection of mouse plasma samples.

NOD-Rag1^–/–^ IL2Rγ mice obtained from The Jackson Laboratory were injected subcutaneously with 3 × 10^5^ cells in 100 μL of cell suspension into the right flank. Terminal blood collection was performed through intracardiac puncture, followed by cervical dislocation, when tumor volume reached 1,500 mm^3^. Ketamine (100 mg/kg) was injected intraperitoneally to induce deep anesthesia (confirmed by toe pinch). Approximately 1 mL of blood was collected into an EDTA tube and immediately placed on ice. Blood was processed by centrifuging at 2,500*g* at 4°C for 10 minutes and 16,100*g* at 4°C for 10 minutes. Plasma was aliquoted stored at −80°C until use.

### DNA-IP and purification.

dsDNA was isolated from conditioned media and human plasma by IP. First, anti-dsDNA antibody (ab27156; mouse monoclonal; Abcam) or normal mouse IgG (MilliporeSigma) was coupled to magnetic beads using the Dynabeads Antibody Coupling Kit (Thermo Fisher Scientific) according to the manufacturer’s instructions. For each IP reaction, 40 μL of antibody-coupled beads (10 mg/mL) was mixed with 100 μL conditioned media or plasma and incubated on a roller for 1 hour at room temperature. Samples were then placed on a magnet rack and the supernatant was collected. Pellets were washed once with PBS and resuspended in PBS. DNA was purified using the DNeasy Blood and Tissue Kit (Qiagen) according to manufacturer’s instructions and kept at 4°C for immediate use or stored at –20°C long-term.

### Permeabilization/degradation assays.

For the HCT116 cell positive control, 5 × 10^4^ cells were suspended in 100 μL PBS for each treatment group. For conditioned media and plasma samples, 100 μL of sample for each treatment group was pretreated with 1 μL Triton X-100 (Thermo Fisher Scientific) or PBS and incubated for 10 minutes at room temperature. Next, samples were treated with 2 U DNase I (New England Biolabs) or PBS, along with 1× DNase I Reaction Buffer (10 mM Tris-HCl, 2.5 mM MgCl_2_, 0.5 mM CaCl_2_, pH 7.6; New England Biolabs) and incubated for 30 minutes at 37°C. The DNase I reaction was halted by adding 1 μL of 0.5 M EDTA and incubating for 10 minutes at 75°C. For plasma samples, the heat inactivation step was omitted (as heating increased plasma viscosity, making efficient DNA-IP difficult), and 3 μL of 0.5 M EDTA was added instead to halt DNase I activity. Treated samples were then subjected to IP and DNA purification as described above.

### Bulk DNA quantification.

For experiments in which purified genomic DNA was subjected to DNA-IP, bulk DNA quantification was performed using the Qubit dsDNA high-sensitivity assay (Invitrogen, Thermo Fisher Scientific). The Qubit assay was conducted using a Life Technologies (Thermo Fisher Scientific) Qubit 3.0 Fluorimeter according to the manufacturer’s instructions.

### nDNA quantification.

nDNA was quantified by qPCR using a Bio-Rad CFX96 Touch Real-Time PCR Detection System. The qPCR assay targeted the second ORF of *LINE1*, a retrotransposon sequence with approximately 100,000 repetitive elements dispersed throughout the genome. *LINE1* has an extremely high copy number that allows for highly sensitive detection of nDNA (29). Standard curves were generated using known concentrations of Human Male or Mouse Genomic DNA (Promega). For human samples, nDNA was quantified by targeting short human *LINE1* for human samples and short mouse *Line1* for murine samples; primer sequences are provided in Supplemental Table 3. Primers were obtained from Integrated DNA Technologies. Species specificity was confirmed for primer pairs prior to use. PCR conditions were as follows: DNA polymerase activation at 95°C for 3 minutes, followed by 40 cycles of denaturation at 95°C for 10 seconds and annealing/extension at 55°C for 30 seconds. The sample volume for each reaction was 10 μL, and a melt curve was included with each run to ensure a single peak with no off-target amplification. A DNA-free negative control was included with each run to ensure that all samples were above the minimum threshold of detection for this assay.

### EV isolation by differential ultracentrifugation.

Conditioned media were collected from HCT116 cells seeded at 8 × 10^5^ cells per plate in 10 cm plates after 48 hours’ incubation. Conditioned media were first centrifuged at 300*g* for 10 minutes; the supernatant from this spin was collected and subsequently centrifuged at 2,000*g* for 20 minutes. Again, the supernatant from this spin was collected and centrifuged at 15,000*g* for 30 minutes. The supernatant was then collected and passed through a 0.2 μm filter, followed by ultracentrifugation at 100,000*g* for 2 hours. The resulting supernatant was collected at stored at 4°C for short-term use and –80°C for long-term storage. The pellet from this ultracentrifugation step was gently rinsed 2 times with ice-cold PBS and resuspended in PBS. The resuspended pellet was stored at –80°C. The 300*g* and 2,000*g* spins were performed using an Eppendorf 5810R centrifuge, while the higher-speed spins were performed using a Beckman Coulter Optima XPN-80 ultracentrifuge. All spins were conducted at 4°C.

### Lipid quantification.

Lipid content associated with dsDNA in conditioned media and plasma samples was determined by a modified sulfo-phospho-vanillin assay, as described elsewhere (36). Briefly, samples were subjected to DNA-IP as described above. Supernatants were collected and diluted, while pellets were treated with Proteinase K (QIAGEN) for 30 minutes at 37°C to remove beads. Next, 200 μL of 96% sulphuric acid was added to 40 μL of each sample, followed by brief vortex and incubation on a heat block at 90°C for 20 minutes. After being allowed to cool, 120 μL phospho-vanillin reagent (0.1% vanillin) (MilliporeSigma) in 17% phosphoric acid (Cedarlane Laboratories) was added to each sample. Finally, samples were transferred to a clear-bottom, 96-well plate for 1 hour at 37°C, and absorbance was measured at 540 nm using a BMG Labtech CLARIOstar Plus plate reader. Sample lipid concentrations were calculated according to a 1,2-dioleoyl-*sn*-glycero-3-phosphocholine (MilliporeSigma) standard curve.

### Immunoblotting of EV markers.

Concentrated conditioned media samples were subjected to Western blot to visualize relevant protein markers. First, IP was performed on samples as described above. A Bradford assay was conducted to determine protein concentrations, and 5 μg of each sample was run on an AnykD Mini-PROTEAN TGX Pre-Cast Gel (Bio-Rad) at 135 V for 50 minutes. Protein was transferred to a PVDF membrane using a Bio-Rad Trans-Blot Turbo. Membranes were blocked in 5% skim milk for 1 hour at room temperature and incubated with the following primary antibodies overnight at 4°C: anti-HSP90 (37-9400; mouse monoclonal; Thermo Fisher Scientific), anti-CD63 (25682-1-AP; rabbit polyclonal; Thermo Fisher Scientific), anti-flotillin1 (ab133497; rabbit monoclonal; Abcam), anti-syntenin1 (ab19903; rabbit polyclonal; Abcam), and anti–histone H3 (ab1791; rabbit polyclonal; Abcam); these markers were selected based on the Minimal Information for Studies of Extracellular Vesicles 2018 guidelines (19) and a recent comprehensive overview of EV protein markers (53). Following primary antibody incubation, membranes were washed in 1× Tris-buffered saline with Tween 20 (TBS-T) and incubated with the corresponding IRDye 800CW goat anti-mouse or anti-rabbit secondary antibody (LI-COR Biosciences) for 1 hour at room temperature. Membranes were subsequently washed in 1× TBS-T and imaged on a LI-COR Odyssey CLx.

### CD9 IP.

CD9^+^ EVs were isolated from conditioned media by IP. First, anti-CD9 antibody (ab263019; rabbit monoclonal; Abcam) was coupled to magnetic beads using the Dynabeads Antibody Coupling Kit (Thermo Fisher Scientific) according to the manufacturer’s instructions. For each IP reaction, 50 μL (for downstream immunoblotting) or 100 μL (for downstream lipid quantification) of antibody-coupled beads was mixed with 140 μL conditioned media and incubated on a roller for 1 hour at room temperature. Samples were then placed on a magnet rack and the supernatant was collected. Pellets were washed once with PBS and resuspended in PBS. Samples were then subjected to downstream assays, as previously specified.

### DNA fragment size analysis and DII.

First, fragment lengths of purified cfDNA from HCT116-conditioned media and DNA-IP pellet were quantified using an Agilent TapeStation 2200 to ensure consistency in immunoprecipitated DNA structure. Next, HCT116-conditioned were was subjected to either no treatment or digestion with 20 U/mL of MNase for 30 minutes at 37°C to degrade exposed DNA linker regions. After treatment, cfDNA was purified from each sample, and fragment lengths were quantified using an Agilent BioAnalyzer 2100. TapeStation data were analyzed using TapeStation Software (Version A.02.02; Agilent), and BioAnalyzer data were analyzed using 2100 Expert software (Version B.02.08.SI648; Agilent).

For fragment size analysis of human plasma subjected to DNA-IP, cfDNA from the input and DNA-IP pellet fractions was measured using an Agilent BioAnalyzer 2100. To determine DII of plasma cf-nDNA, purified DNA samples were subjected to qPCR using primer sets targeting short and long human *LINE1*; primer sequences are provided in Supplemental Table 3. DII was calculated by dividing the amount of long *LINE1* by the amount of short *LINE1*. Therefore, smaller overall fragment size is reflected by a lower DII (as a DII of 1 would imply fragment lengths ≥ 224 bp).

### mtDNA quantification.

mtDNA from all samples was quantified by ddPCR using the QX200 Droplet Digital PCR System (Bio-Rad). For human samples, mtDNA was quantified by targeting *MTND1* as described elsewhere (66). For murine samples, mtDNA was quantified by targeting mouse *Mtnd1*; primers were designed with Primer3Plus. All primers and primer/probe sets were obtained from Integrated DNA Technologies, and sequences are provided in Supplemental Table 3. mtDNA primers were validated by qPCR on purified human and mouse mtDNA isolated from HCT116 and MC38 cells, respectively, using a mitochondrial DNA isolation kit (Abcam), as per the manufacturer’s instructions. Next, an 8-step ddPCR temperature gradient (54°C−64°C) was conducted to determine optimal amplification conditions (56°C for human *MTND1*; 58°C for mouse *Mtnd1*). Subsequently, purified DNA samples were run on ddPCR according to the manufacturer’s instructions. A standard containing relevant DNA template was included in each run, and the signal threshold was set according to this standard. A blank was also included in each run to preclude sample contamination. Only samples with >12,000 events were included in analyses, as per Bio-Rad’s suggested protocols. Data were analyzed using QuantaSoft Analysis Software (Version 1.7.4.0917; Bio-Rad).

### ctDNA quantification.

ctDNA from HPV^+^ HNC and AML patients was also quantified by ddPCR. For HPV^+^ HNC samples, tumor-derived nDNA was quantified by targeting both HPV16 E6 and E7 in separate assays and averaging the copy number from both assays for each sample (67). For AML samples, *NPM1* DNA was amplified using a single primer set for both wild-type and type A mutant, and unique probes were then used to quantify wild-type and type A mutant. These primer/probe sets were designed using Primer3Plus and obtained from Integrated DNA Technologies; primer and probe sequences are provided in Supplemental Table 3. HPV-specific primers were validated by qPCR using purified genomic DNA from the HPV^+^ SiHa cell line (ATCC). Primers for *NPM1* were validated by qPCR using DNA oligos containing either the wild-type or type A mutant *NPM1* sequence. ddPCR assays for all HNC and AML primer/probe sets were run at 56°C and 60.2°C, respectively, as determined by an 8-step ddPCR temperature gradient. Samples were run and analyzed as described in the *mtDNA quantification* section above.

### EV inhibition assays.

On day 0, 2 × 10^5^ HCT116 cells were seeded in each well of a 6-well plate in normal media, as described above. After 24 hours (i.e., day 1), media were aspirated from each well and cells were rinsed twice with PBS. Next, cells were cultured in either vehicle-containing media (Y-27632: H_2_O; GW4869: DMSO), or inhibitor-containing media (1 μM Y-27632 or 10 μM GW4869) and incubated for 24 hours. On day 2, conditioned media were collected and spun using an Eppendorf 5810R centrifuge at 301*g* for 5 minutes at 4°C to remove cells.

DNA was purified from each conditioned media sample, and nDNA and mtDNA content were quantified by qPCR and ddPCR, respectively, as described previously. Particle concentration and size were determined by NTA using a Malvern Panalytical NanoSight N300. Prior to NTA, conditioned media were subjected to buffer exchange with Amicon Ultra-2 Centrifugal Filter Devices with a 3 kDa MW cutoff (MilliporeSigma) using an Eppendorf 5810R centrifuge at 3,180*g* for 50 minutes at 15°C, followed by a recovery spin at 1,000*g* for 2 minutes at 15°C. The resulting concentrate was brought back up to its original volume with PBS.

### Histone IP and immunoblotting.

Concentrated conditioned media from inhibitor-treated cells were subjected to histone IP using anti-histone antibody (MAB3422; mouse monoclonal; MilliporeSigma) conjugated to Dynabeads (Thermo Fisher Scientific), as described above. For each IP reaction, 40 μL of antibody-coupled beads (10 mg/mL) was mixed with 100 μL concentrated media and incubated on a roller for 1 hour at room temperature. Samples were then placed on a magnet rack and the supernatant was discarded. Pellets were washed once with PBS and resuspended in 25 μL of 2× loading buffer (Bio-Rad). Western blot was performed as described above using the primary antibody anti–histone H3 (ab1791; rabbit polyclonal; Abcam). Band intensity was quantified using Image Studio Lite software (version 5.2, LI-COR Biosciences).

### Apoptosis activity assay.

To start, 8 × 10^3^ HCT116 cells were seeded in a white-walled, clear-bottom, 96-well plate in 200 μL of inhibitor-free media. After 24 hours, cells were treated with either vehicle or inhibitor, as described above; Z-VAD-FMK (20 μM; InvivoGen) was included as a control for inhibition of apoptosis. After another 24 hours’ incubation, 20 μL media was removed from each well, and 20 μL PrestoBlue Cell Viability Reagent (Thermo Fisher Scientific) was added. Cells were incubated for 1 hour at 37°C, and fluorescence was measured according to manufacturer’s recommendations using a BMG Labtech CLARIOstar Plus plate reader. Next, 100 μL media was replaced with 100 μL Caspase-Glo 3/7 reagent (Promega), and cells were incubated in the dark for 1 hour at room temperature; luminescence was measured according to manufacturer’s recommendations using a BMG Labtech CLARIOstar Plus plate reader. Caspase activity was normalized to cell viability within each treatment to determine the normalized caspase activity as a measure of apoptosis.

### Statistics.

For cell line experiments, data were obtained from technical triplicates from the same biological source, unless otherwise stated. Human and xenograft plasma measurements were conducted on distinct biological sources, and sample size for each cohort is stated in the main text and in figure legends. Unpaired *t* test or Mann-Whitney *U* test was used to analyze statistical significance between 2 groups. Two-way ANOVA was employed when assessing statistically significant differences between groups affected by 2 factors (i.e., treatments). For ANOVAs, Tukey’s multiple-comparison test was used to determine significant differences between each group. Pearson’s correlation and Spearman’s correlation were used to assess relationships between 2 variables for linear and nonlinear regressions, respectively. For recurrence-free survival analysis, events were defined by disease recurrence (deaths in the absence of documented disease recurrence were censored) and measured from the time of diagnosis; groups of patients were compared using the log-rank test. All tests were performed as 2 sided unless otherwise stated. Statistical significance was determined using a *P* value less than 0.05. All statistical analyses were performed using Prism software (version 9.0.2, GraphPad Software LLC). All data are reposted as mean ± SD unless otherwise stated.

### Study approval.

All studies involving human specimens were approved by the Research Ethics Board at University Health Network (Toronto, Ontario, Canada). Informed consent was obtained from all human participants prior to participation. Animal experiments were performed with the approval of the University Health Network Animal Care Committee (Toronto, Ontario, Canada) and adhered to the Canadian Council on Animal Care guidelines (protocol 4051).

## Author contributions

SVB and EZM conceived the project; EZM, SDM, ML, and ZZ performed experimental design; AA, MDM, and SVB performed sample acquisition; EZM, SD, ML, and RG performed data acquisition; EZM, SDM, and ZZ performed data analysis; EZM wrote the first draft; EZM, SDM, ML, RG, AR, MDM, and SVB made revisions and edits to the draft; SVB supervised the project; and SVB acquired funding.

## Supplementary Material

Supplemental data

## Version 1 09/20/2022

In-Press Preview

## Version 2 10/24/2022

Electronic publication
