# Supplementary material for: Cell-free DNA topology depends on its subcellular and cellular origins in cancer
Source: JCI Insight. 2022 Oct 24;7(20):e159590. doi: 10.1172/jci.insight.159590 (PMC9714790; doi:10.1172/jci.insight.159590)

**SUPPLEMENTARY INFORMATION**

# TITLE: Cell-free DNA topology depends on its subcellular and cellular origins in cancer

**Ethan Z. Malkin[1,2], Steven De Michino[1,2], Meghan Lambie[1,2], Rita Gill[2], Zhen Zhao[2], Ariana Rostami[2], Andrea Arruda[2], Mark D. Minden[2], and Scott V. Bratman[1,2,3]***

[1]Department of Medical Biophysics, Temerty Faculty of Medicine, University of Toronto, Toronto, Ontario, Canada

[2]Princess Margaret Cancer Centre, University Health Network, Toronto, Ontario, Canada

[3]Department of Radiation Oncology, Temerty Faculty of Medicine, University of Toronto, Toronto, Ontario, Canada

## SUPPLEMENTARY TABLES AND FIGURES

| | Input | | Pellet | | Supernatant | | Relative |
|---|---|---|---|---|---|---|---|
| | nDNA Concentration (ng/mL) | nDNA Amount (ng) | nDNA Concentration (ng/mL) | nDNA Amount (ng) | nDNA Concentration (ng/mL) | nDNA Amount (ng) | Recovery (%) |
| **Purified gDNA** | | | | | | | |
| Qubit dsDNA HS | 18.733 ± 0.1530 | 1.873 ± 0.0153 | 14.133 ± 0.379 | 1.413 ± 0.080 | *n.d.* | *n.d.* | 100.0 ± 0.000 |
| LINE1 qPCR | 22.278 ± 1.475 | 2.228 ± 0.148 | 20.142 ± 0.686 | 2.014 ± 0.069 | 3.96E-4 ± 5.14E-5 | 3.96E-5 ± 5.14E-6 | 99.99 ± 0.000 |
| **Cell Lines** | | | | | | | |
| MCF10A | 14.280 ± 1.060 | 1.428 ± 0.106 | 10.480 ± 0.182 | 1.048 ± 0.018 | 0.040 ± 0.002 | 0.0004 ± 0.0002 | 99.97 ± 0.001 |
| MC38 | 3.514 ± 1.127 | 0.351 ± 0.113 | 4.020 ± 1.707 | 0.402 ± 0.171 | 0.004 ± 0.001 | 0.0004 ± 0.0001 | 99.88 ± 0.026 |
| A549 | 1.275 ± 0.182 | 0.128 ± 0.018 | 1.388 ± 0.152 | 0.139 ± 0.015 | 0.003 ± 0.001 | 0.0003 ± 0.0001 | 99.77 ± 0.089 |
| Cal33 | 2.221 ± 0.047 | 0.222 ± 0.005 | 1.950 ± 0.054 | 0.195 ± 0.005 | 0.038 ± 0.002 | 0.0038 ± 0.0002 | 98.11 ± 0.151 |
| KYSE410 | 28.677 ± 1.321 | 2.868 ± 0.132 | 20.520 ± 1.257 | 2.052 ± 0.126 | 0.405 ± 0.021 | 0.0405 ± 0.0021 | 98.06 ± 0.176 |
| HCT116 | 82.963 ± 10.755 | 8.296 ± 1.076 | 79.697 ± 7.939 | 7.970 ± 0.794 | 1.364 ± 0.121 | 0.1364 ± 0.0121 | 95.36 ± 0.984 |
| SU-DHL-6 | 34.168 ± 2.679 | 3.417 ± 0.268 | 28.63 ± 6.969 | 2.863 ± 0.670 | 1.571 ± 0.067 | 0.1571 ± 0.0067 | 94.65 ± 0.967 |
| B16F10 | 3.799 ± 0.817 | 0.380 ± 0.082 | 2.713 ± 0.534 | 0.271 ± 0.053 | 0.185 ± 0.026 | 0.0185 ± 0.0026 | 93.46 ± 1.556 |
| **Human Plasma** | | | | | | | |
| Healthy Donor | 38.586 ± 99.934 | 3.859 ± 9.993 | 14.602 ± 10.475 | 1.460 ± 1.048 | 0.363 ± 0.917 | 0.0363 ± 0.0917 | 97.72 ± 4.663 |
| HPV+ HNC | 27.029 ± 41.938 | 2.703 ± 4.194 | 18.511 ± 18.076 | 1.851 ± 1.808 | 0.173 ± 0.308 | 0.0173 ± 0.0308 | 99.01 ± 1.662 |
| HPV- HNC | 29.976 ± 113.246 | 2.998 ± 11.325 | 14.574 ± 9.096 | 1.457 ± 0.910 | 0.257 ± 1.076 | 0.0257 ± 0.1076 | 98.87 ± 2.497 |
| AML | 213.72 ± 215.727 | 21.372 ± 21.573 | 1320.558 ± 1719.099 | 132.056 ± 171.910 | 2.630 ± 2.659 | 0.2630 ± 0.2659 | 99.61 ± 0.239 |
| **Xenograft Plasma** | | | | | | | |
| Host | 3.789 ± 5.200 | 0.379 ± 0.520 | 3.854 ± 5.167 | 0.385 ± 0.517 | 0.069 ± 0.112 | 0.0069 ± 0.0011 | 97.43 ± 4.844 |
| Tumour | 7.597 ± 7.334 | 0.760 ± 0.733 | 8.302 ± 8.534 | 0.830 ± 0.853 | 0.032 ± 0.019 | 0.0032 ± 0.0019 | 98.45 ± 4.029 |

**Supplementary Table 1. Absolute quantities of cf-nDNA in DNA-IP fractions from purified genomic DNA control, cell line, and plasma samples.** The absolute quantity in each fraction was determined by multiplying the concentration by the volume of the starting DNA-IP sample, which was 0.1mL in all experiments. The input fraction refers to media or plasma not subjected to DNA-IP. The relative recovery in the DNA-IP pellet was determined by dividing the amount of nDNA in the pellet fraction by the sum of nDNA in the pellet and supernatant fractions. nDNA concentration was quantified by species-specific *LINE1* qPCR unless otherwise specified. Data are reported as mean ± SD. *n.d.* = not detectable.

| | Cohort | | | |
|---|---|---|---|---|
| | **HD** | **HPV+ HNC** | **HPV- HNC** | **AML** |
| **Total Cases** | 50 | 49 | 44 | 6 |
| **Mean Age** | 59.9 | 60.6 | 60.8 | 78.0 |
| **Sex** | | | | |
| Male | 38 | 44 | 38 | 5 |
| Female | 6 | 5 | 6 | 1 |
| **Stage** | | | | |
| Early (I-II) | - | 37 | 12 | - |
| Late (III-IV) | - | 12 | 32 | - |

**Supplementary Table 2. Demographic and clinical information of healthy donor and cancer patient cohorts.** Age and sex data was not available for 6 healthy donors because these 6 samples were commercially purchased. Staging was not available for the AML cohort, as white blood cell and blast counts were recorded at the time of sample collection in lieu of stage information.

| Target | Sequence (5’-3’) | Amplicon (bp) |
|---|---|---|
| Short human *LINE1* | Fwd: TCTGCCTTCATTTCGTTATGTACC<br>Rev: TCACTCAAAGCCGCTCAACTAC | 82 |
| Long human *LINE1* | Fwd: TCTGCCTTCATTTCGTTATGTACC<br>Rev: TCAGCACCACACCACACCTATTC | 224 |
| Short mouse *Line1* | Fwd: AATGGAAAGCCAACATTCACGTG<br>Rev: CCTTCCTTGACCAAGGTATCATTG | 71 |
| Human *MTND1* | Fwd: CCCTAAAACCCGCCACATCT<br>Rev: GAGCGATGGTGAGAGCTAAGGT<br>Probe: (FAM)-CCATCACCCTCTACATCACCGCCC | 69 |
| Mouse *Mtnd1* | Fwd: ACTCCTCGTCCCCATTCTAA<br>Rev: AATGCCGTATGGACCAACAA<br>Probe: (FAM)-AGAACGCAAAATCTTAGGGTACATACAA | 112 |
| HPV16 E6 | Fwd: ACTGTGTCCTGAAGAAAAGCA<br>Rev: GTCCACCGACCCCTTATATT<br>Probe: (FAM)-ACATCTGGACAAAAAGCAAAGATTCCA | 72 |
| HPV16 E7 | Fwd: GAGGAGGATGAAATAGATGGTC<br>Rev: CCGAAGCGTAGAGTCACA<br>Probe: (HEX)-TGGACAAGCAGAACCGGACA- | 99 |
| *NPM1* WT | Fwd: AGATGTTGAACTATGCAAAGAGACA<br>Rev: TCTTAAAGAGACTTCCTCCACTG<br>Probe: (HEX)-TCCAGGCTATTCAAGATCTCTGG | 132 |
| *NPM1* mutant A | Fwd: AGATGTTGAACTATGCAAAGAGACA<br>Rev: TCTTAAAGAGACTTCCTCCACTG<br>Probe: (FAM)-TCCAGGCTATTCAAGATCTCTGTCTGG | 136 |

**Supplementary Table 3. PCR primer and probe sequence and amplicon information.** More information on primer design, validation, and use is available in the *Methods* section of the manuscript.

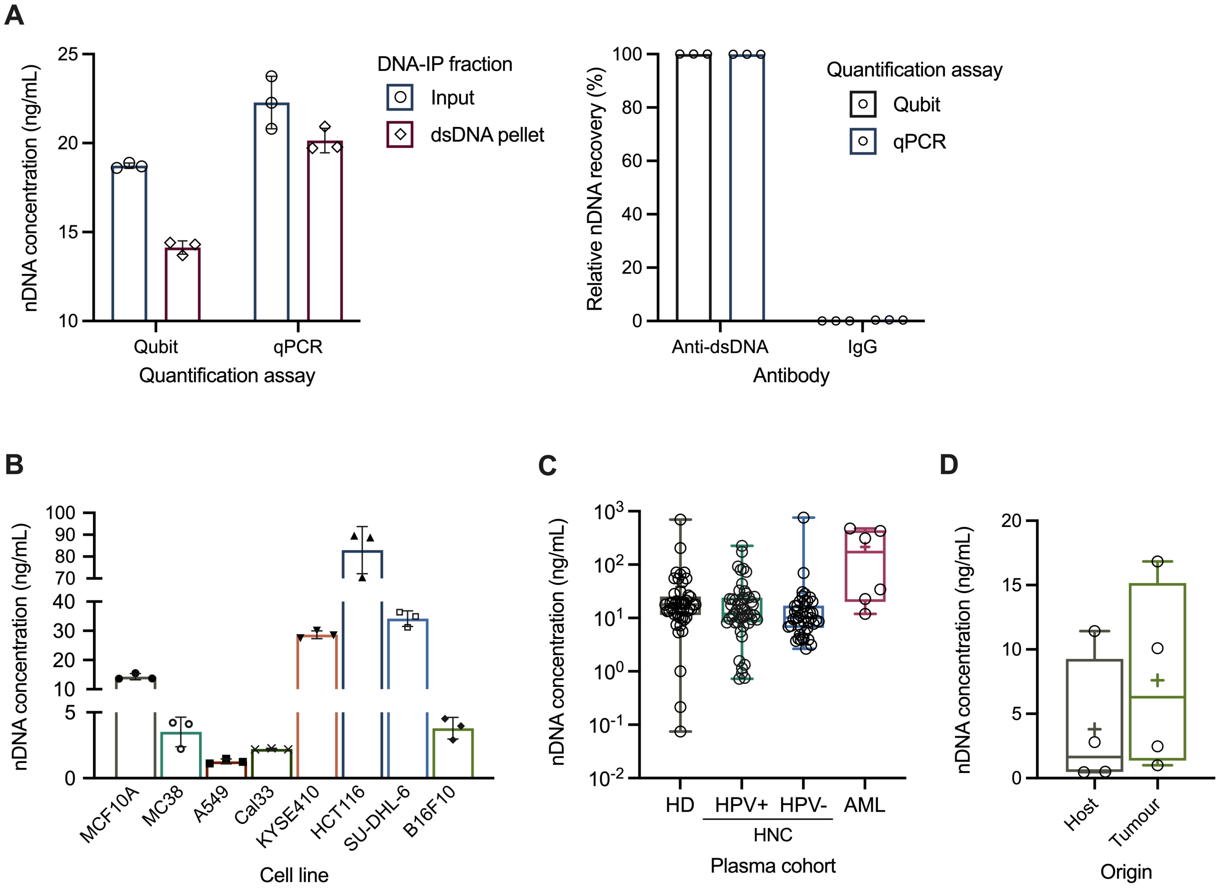


**Supplementary Figure 1. Total cf-nDNA quantification from purified genomic DNA control, cell line, and plasma samples.**

(A) *Left:* Concentration of purified genomic DNA without (i.e., input) and with (i.e., dsDNA pellet) DNA-IP, as quantified by Qubit dsDNA High Sensitivity assay and nDNA-specific short *LINE1* qPCR. *Right:* Relative recovery of purified genomic DNA by DNA-IP.

(B and C) Concentration of cf-nDNA in conditioned media of cell line panel (B) or HD plasma and cancer patient plasma cohorts (C). Data are reported as mean ± SD (B) or as a box-and-whiskers plot (C) displaying both mean (represented by + symbol) and median (represented by horizontal line in box), as well as the minimum (lower whisker), 25$^{th}$ percentile (lower bound of box), 75$^{th}$ percentile (upper bound of box), and maximum (upper whisker).

(D) Concentration of host- and tumour-derived cf-nDNA from Cal33 xenograft plasma. Xenograft host cf-nDNA was quantified by qPCR using mouse-specific *Line1* primers and xenograft tumour cf-nDNA was quantified by qPCR using human-specific *LINE1* primers. Data are reported as a box-and-whiskers plot displaying both mean (represented by + symbol) and median (represented by horizontal line in box), as well as the minimum (lower whisker), 25$^{th}$ percentile (lower bound of box), 75$^{th}$ percentile (upper bound of box), and maximum (upper whisker).

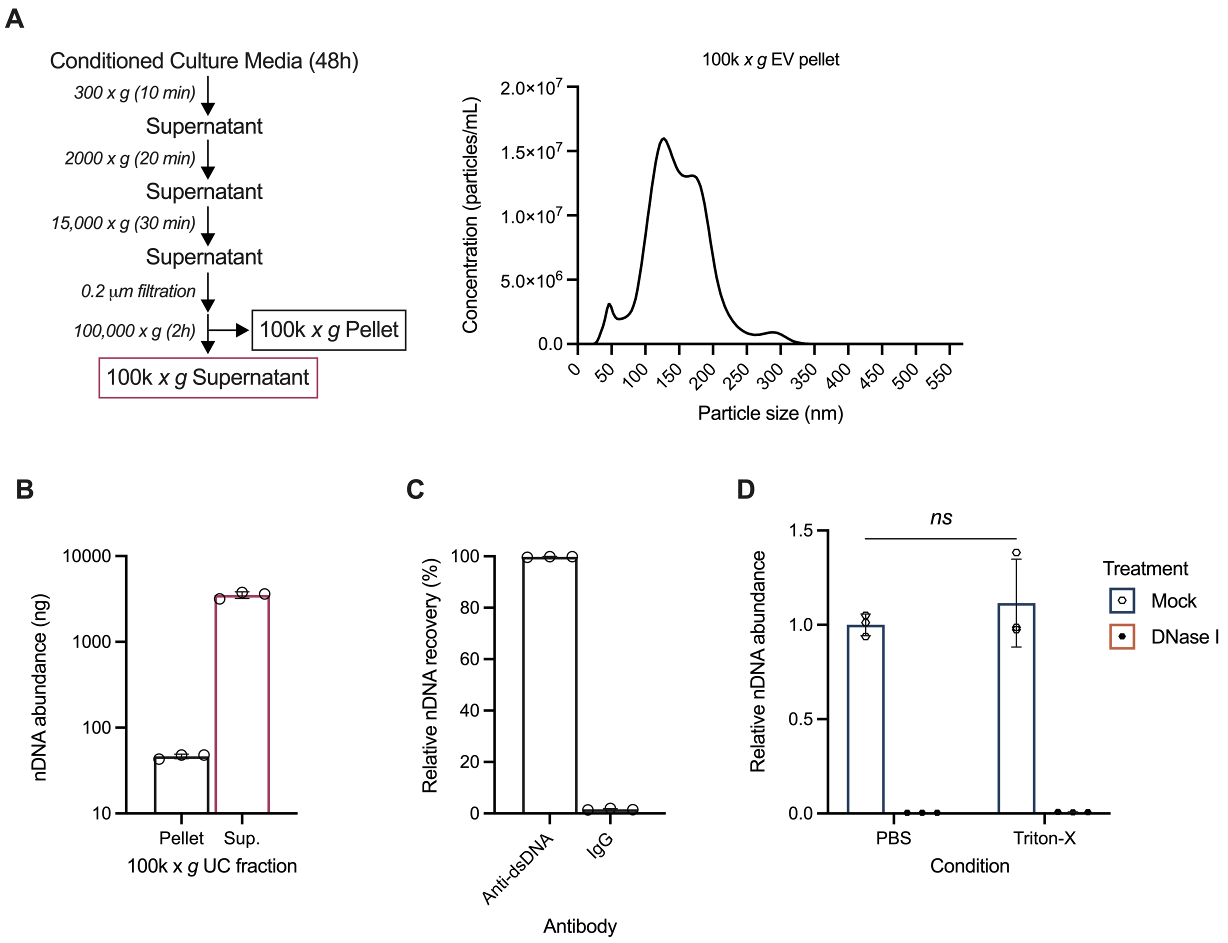


**Supplementary Figure 2. Cell-free nDNA is not membrane-protected in isolated EVs.**

(A) *Left:* schematic of differential ultracentrifugation (UC) protocol used to isolate EVs from conditioned media from HCT116 cells. *Right:* Particle size histogram of EVs isolated by UC from conditioned media from HCT116 cells. Particle sizes were determined by NTA, and particle size is plotted in 1nm bins centered at n+0.5.

(B) Absolute abundance of nDNA in the 100k *x g* UC pellet and supernatant fractions.

(C) Relative recovery of nDNA from the 100k *x g* UC pellet by DNA-IP and IgG control.

(D) Permeabilization/degradation assay on the 100k *x g* UC pellet. Values were normalized to their respective PBS/mock treatment.

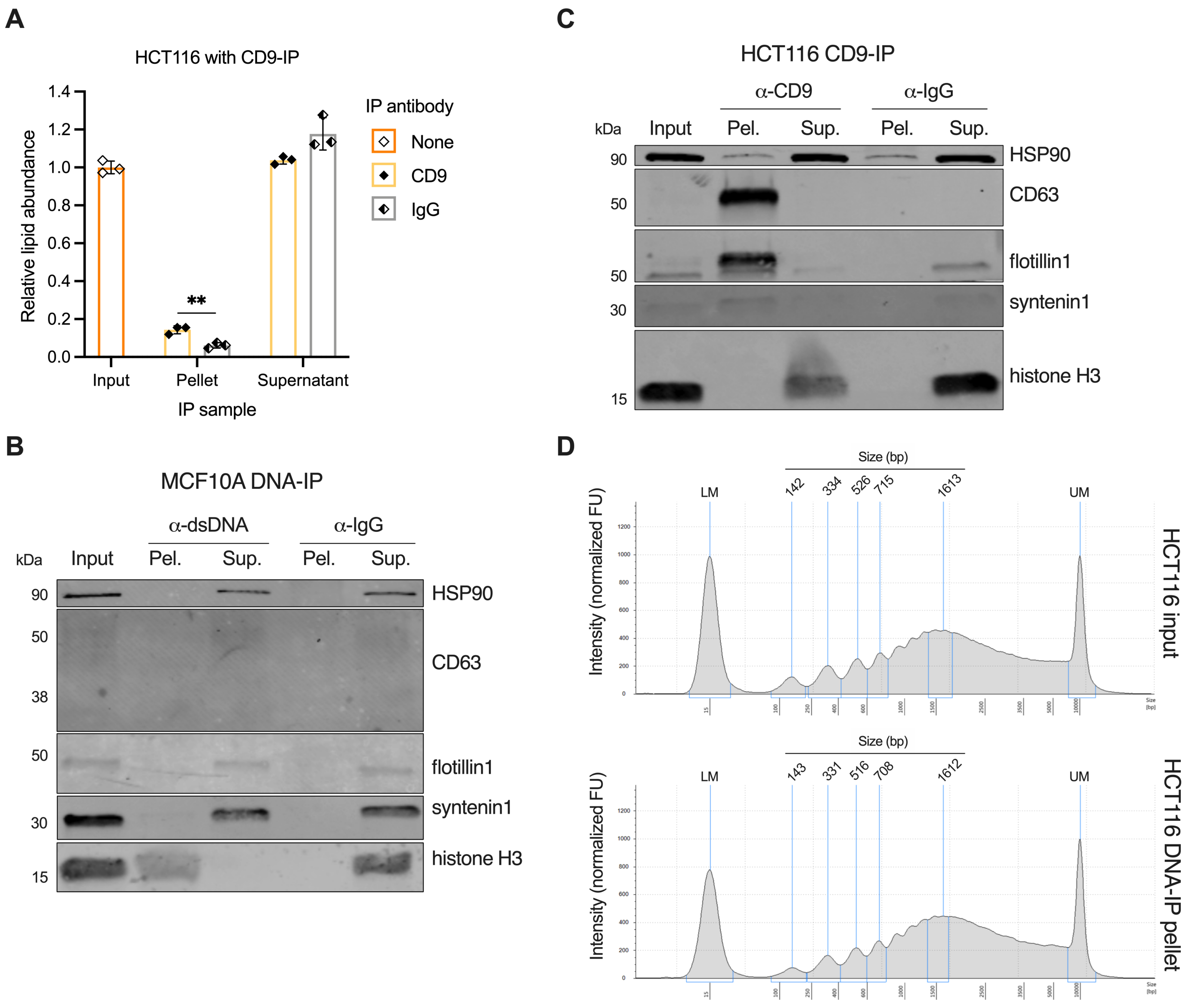


**Supplementary Figure 3. Cell-free DNA structure is similar between cell lines and conserved after DNA-IP.**

(A) Immunoprecipitation of CD9 was performed on conditioned media from HCT116 cells, and lipid content in each fraction was quantified using a modified phospho-sulfo-vanillin assay. Values were normalized to their respective input fraction.

(B and C) Immunoblotting of common EV markers HSP90, CD63, flotillin1, and syntenin1, as well as histone H3, after DNA-IP of MCF10A conditioned media (B) or immunoprecipitation of CD9 on HCT116 conditioned media (C).

(D) Cell-free DNA from conditioned media (top; “input”) or DNA-IP (bottom; “DNA-IP Pellet) was purified, and fragment sizes were quantified by TapeStation. The lower marker (LM) is 15bp and the upper marker (UM) is 10kb. Representative data from HCT116 conditioned media.

*$p$<0.05, **$p$<0.01, ***$p$<0.001, ****$p$<0.0001, ns = not significant; unpaired t test with Welch’s correction (A).

**A**

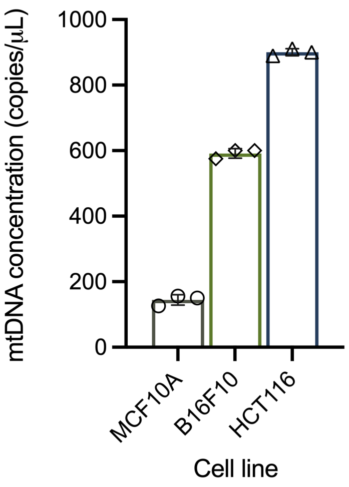


**Supplementary Figure 4. Total cf-mtDNA quantification from cell line samples.**

Concentration of cf-mtDNA in conditioned media of MCF10A, B16F10, and HCT116 cells.

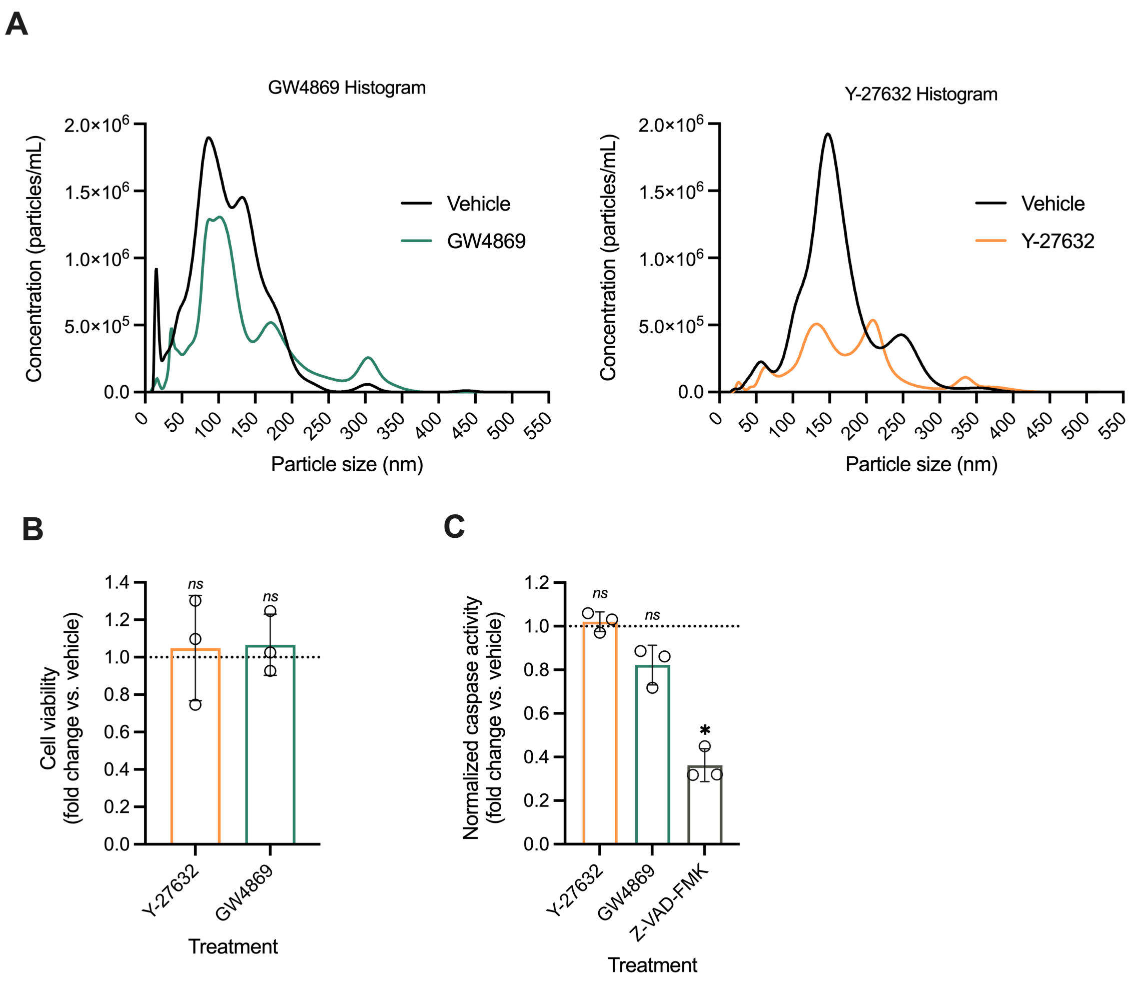


**Supplementary Figure 5. Effects of EV inhibition on particle size and apoptosis.**

(A) Particle size histograms of conditioned media from HCT116 cells treated with Y-27632 (left) or GW4869 (right) or their respective vehicle controls. Particle sizes were determined by NTA, and particle size is plotted in 1nm bins centered at n+0.5.

(B) Viability of cells treated with each inhibitor, as determined by PrestoBlue staining. Values were normalized to the vehicle control.

(C) Normalized caspase activity of HCT116 cells treated with EV inhibitors or the pan-caspase inhibitor Z-VAD-FMK, determined by dividing caspase activity by cell viability within each treatment group. Values were normalized to the vehicle control.

$^{*}p<0.05$, $^{**}p<0.01$, $^{***}p<0.001$, $^{****}p<0.0001$, ns = not significant; unpaired t test (B and C).

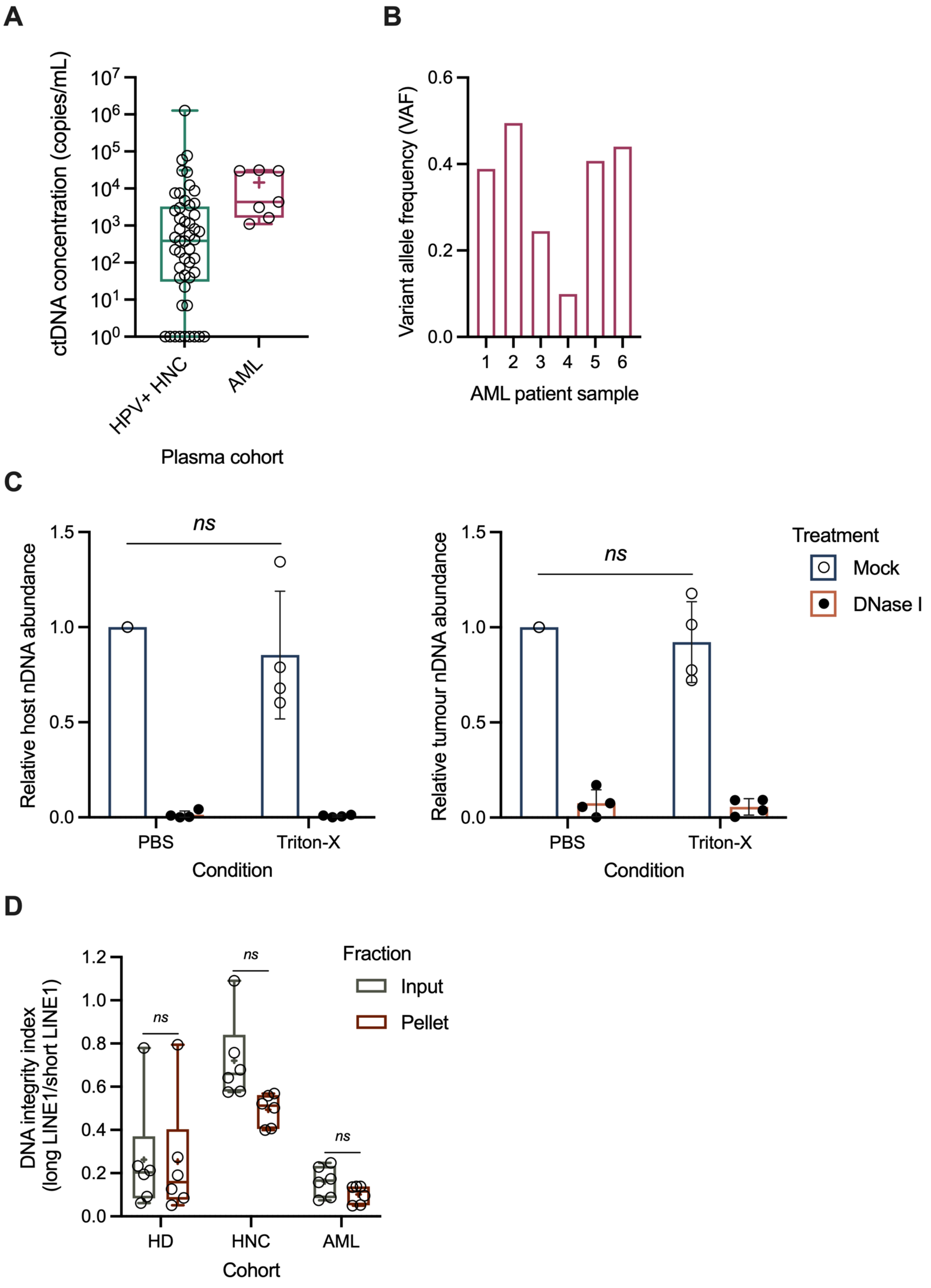


**Supplementary Figure 6. Cell-free nDNA is not EV-associated in cancer patient and xenograft mouse plasma.**

(A) Concentration of total ctDNA in cancer patient plasma. HPV+ HNC tumour DNA was quantified by ddPCR using primers targeting HPV16 E6 and E7 sequences, and total ctDNA was calculated by averaging the E6 and E7 values. AML tumour DNA was quantified by ddPCR using primers targeting the *NPM1* type A mutation. Data are reported as a box-and-whiskers plot displaying both mean (represented by + symbol) and median (represented by horizontal line in box), as well as the minimum (lower whisker), 25$^{th}$ percentile (lower bound of box), 75$^{th}$ percentile (upper bound of box), and maximum (upper whisker).

(B) Variant allele frequency (VAF) in AML patient plasma samples. VAF was determined by dividing the type A mutant *NPM1* copy number by the total *NPM1* copy number (wild-type *NPM1* plus type A mutant *NPM1*). Wild-type and type A mutant *NPM1* copy number was quantified by ddPCR using primers specific to each allele sequence. Data are reported as the mean for each patient sample.

(C) Degradation assays on Cal33 xenograft plasma. Mouse short *Line1* primers were used to quantify cf-nDNA derived from healthy tissue (left), and human short *LINE1* primers were used to quantify tumour-derived cf-nDNA (right), by qPCR. Values were normalized to their respective PBS/mock treatment.

(D) DII of select HD and cancer patient plasma samples. Before (i.e., input) or after DNA-IP (i.e., pellet), DNA was purified and quantified by qPCR using nDNA-specific short and long *LINE1* primers. DII was calculated as the ratio of long *LINE1* amplicon abundance to short *LINE1* amplicon abundance. Data are reported as a box-and-whiskers plot displaying both mean (represented by + symbol) and median (represented by horizontal

line in box), as well as the minimum (lower whisker), 25th percentile (lower bound of box), 75th percentile (upper bound of box), and maximum (upper whisker).

*$p$<0.05, **$p$<0.01, ***$p$<0.001, ****$p$<0.0001, ns = not significant; ordinary two-way ANOVA with Tukey's multiple comparisons test (C and D).

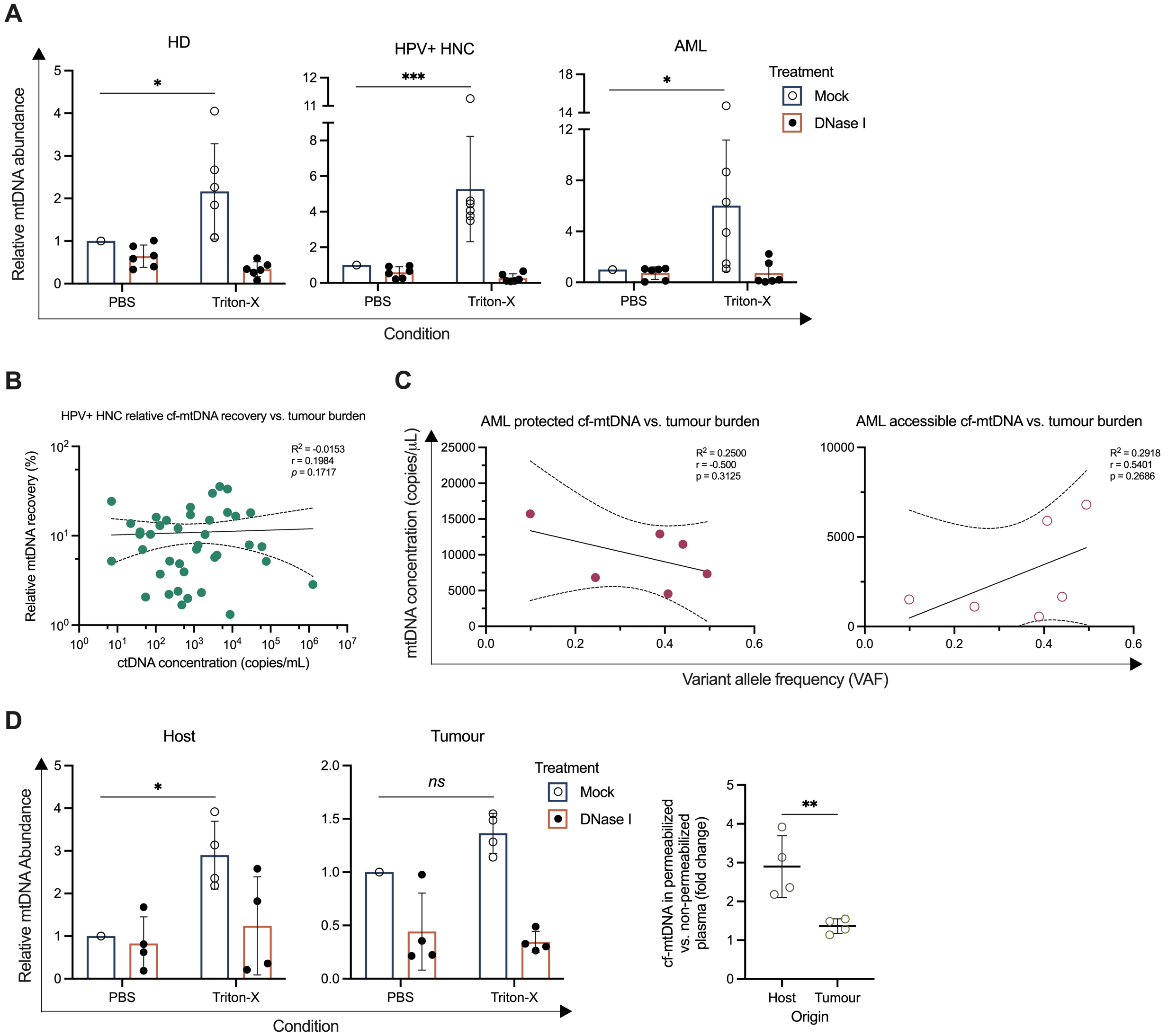


**Supplementary Figure 7. Abundance and protection of plasma cf-mtDNA informs its cellular origins in cancer patients.**

(A) Cell-free mtDNA abundance after permeabilization/degradation assays on select HD, HPV+ HNC, and AML patient plasma. Values were normalized to their respective PBS/mock treatment.

(B) Plot of relative cf-mtDNA recovery in HPV+ HNC patient plasma versus tumour burden (as measured by ctDNA). Data are fit with a non-linear log-log regression and dashed lines represent 95% confidence interval.

(C) Plot of protected (left) and accessible (right) cf-mtDNA in AML (right) patient plasma versus tumour burden (as measured by VAF). Data are fit with a linear regression and dashed lines represent 95% confidence interval.

(D) Cell-free mtDNA abundance after permeabilization/degradation assays on Cal33 xenograft mouse plasma. Human *MTND1* primers were used to quantify tumour-derived mtDNA (left), and mouse *Mtnd1* primers were used to quantify mtDNA derived from healthy tissue (middle), by ddPCR. Values were normalized to their respective PBS/mock treatment. Also shown is the mean fold-change in mtDNA recovery after membrane permeabilization and DNA-IP (right), calculated by dividing the abundance of mtDNA recovered from the permeabilized mock-treated group by the abundance of mtDNA recovered from the non-permeabilized mock-treated group.

*$p<0.05$, **$p<0.01$, ***$p<0.001$, ****$p<0.0001$, ns = not significant; ordinary two-way ANOVA with Tukey's multiple comparisons test (A, D left, and D middle); non-linear log-log regression with Spearman correlation (B); simple linear regression with Pearson correlation (C); unpaired t test (D right). Human cohorts: HPV+ n=49 (n=6 for select samples), AML n=6; n=4 for mouse cohort.

# Full unedited gel images

Malkin *et al.*

Cell-free DNA topology is unique to its sub-cellular and cellular origins in cancer

Full unedited gel for Figure 2D: HSP90, flotillin1, and histone H3

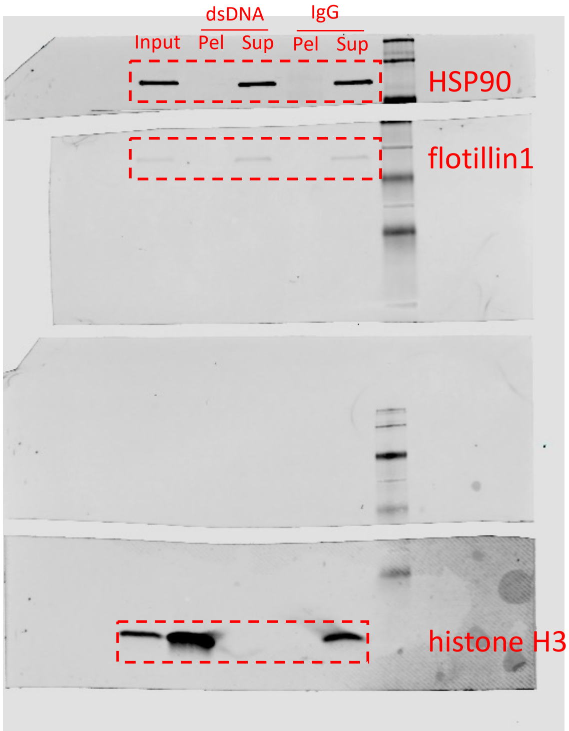

# Full unedited gel for Figure 2D: CD63

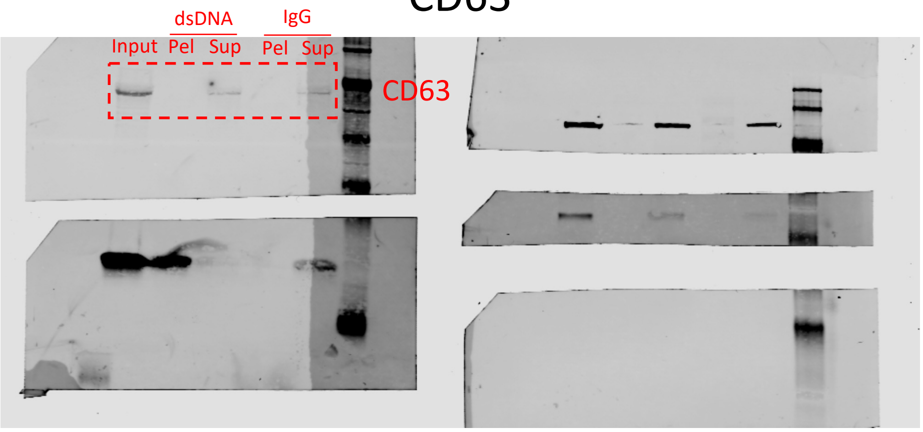


# Full unedited gel for Figure 2D: syntenin1

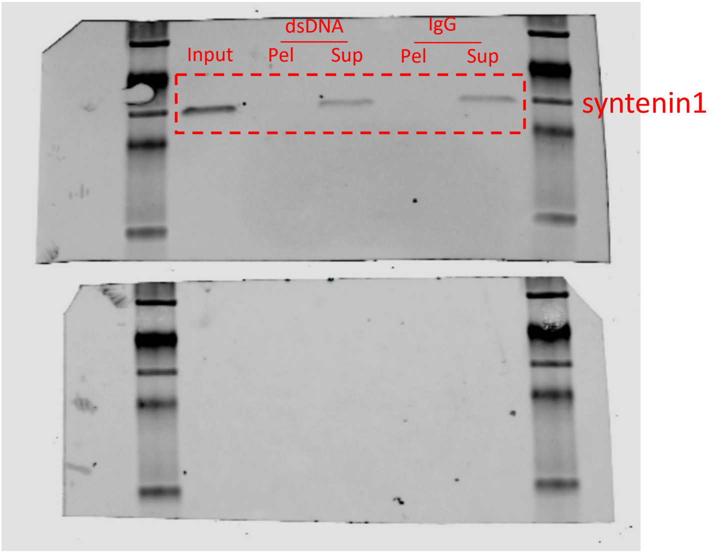

# Full unedited gel for Figure 3G:
# Y-27632

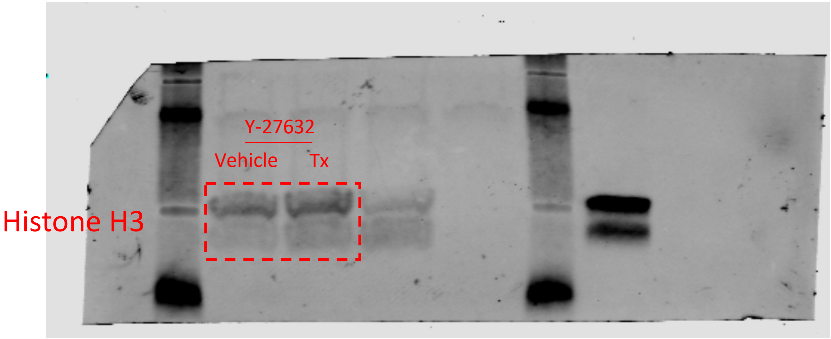


# Full unedited gel for Figure 3G:
# GW4689

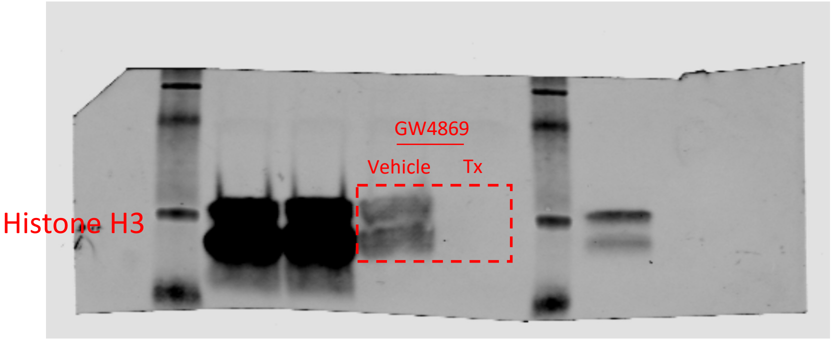

Full unedited gel for Supplementary Figure 3B: HSP90

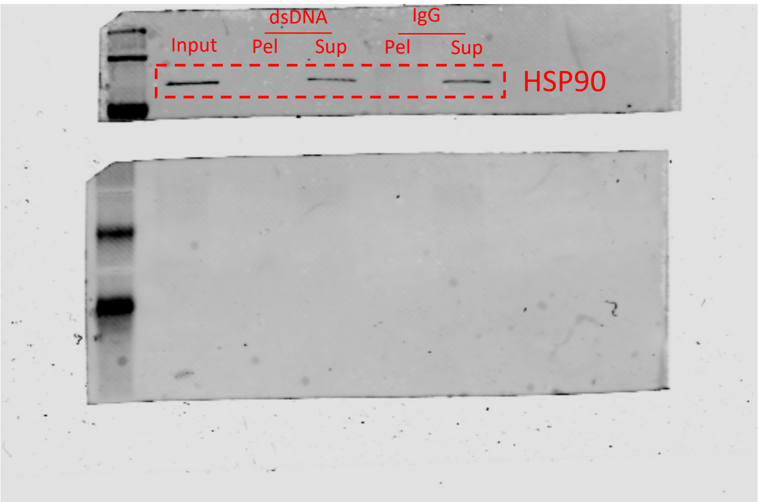


Full unedited gel for Supplementary Figure 3B: CD63

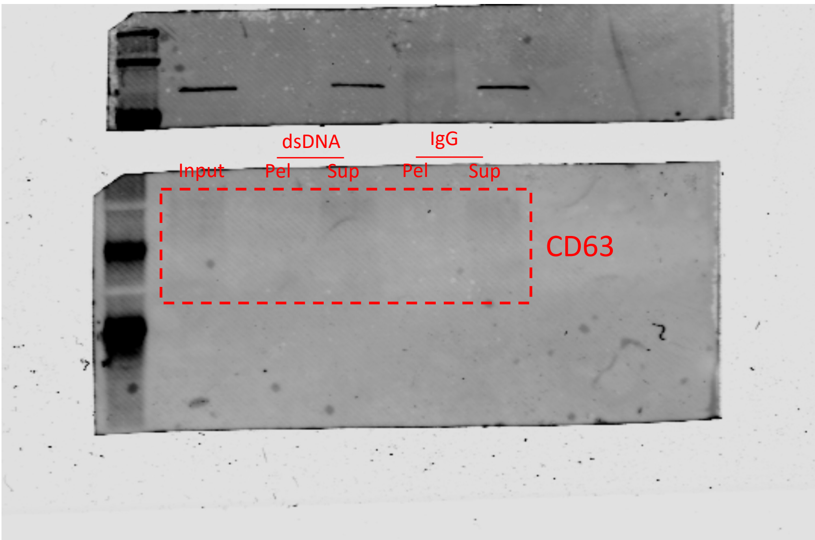

# Full unedited gel for Supplementary Figure 3B: flotillin1

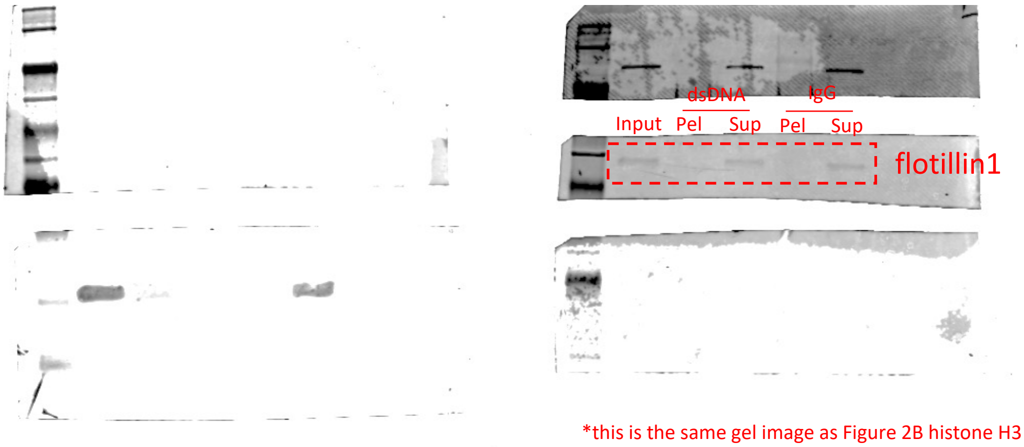


*this is the same gel image as Figure 2B histone H3 (next page), but with higher exposure to better visualize flotillin1 bands. Bands at higher exposure were only compared against each other and not against bands at other exposure levels.

# Full unedited gel for Supplementary Figure 3B: syntenin1

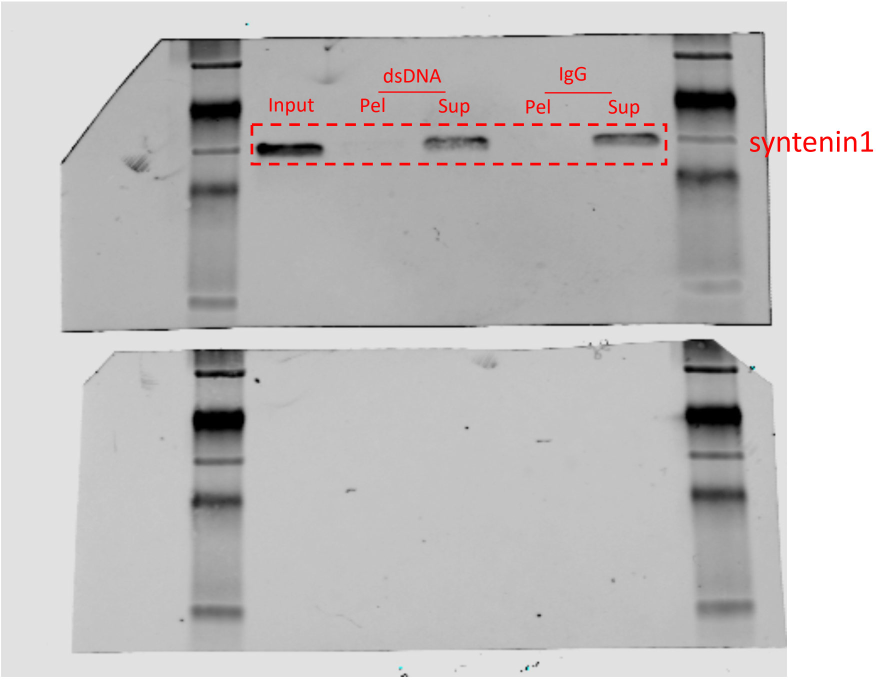

# Full unedited gel for Supplementary Figure 3B: histone H3

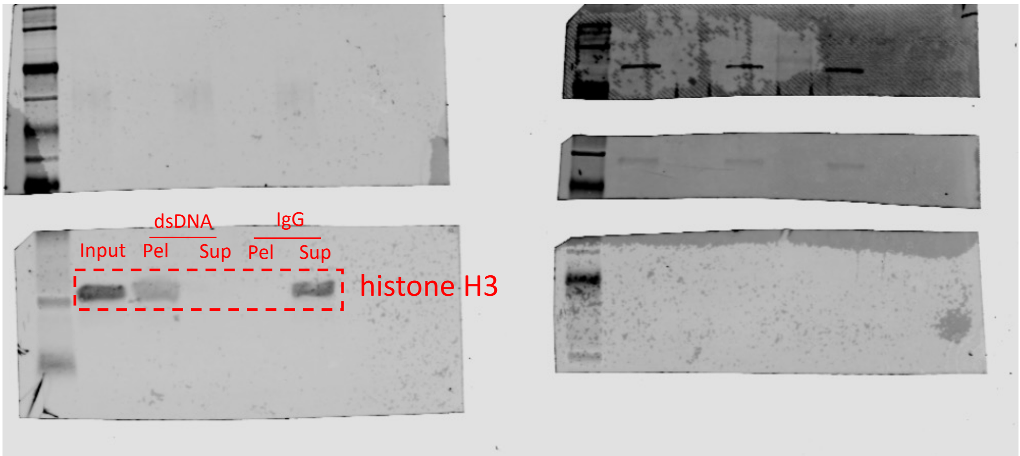

Full unedited gel for Supplementary Figure 3C: HSP90, CD63, flotillin1, syntenin1, and histone H3

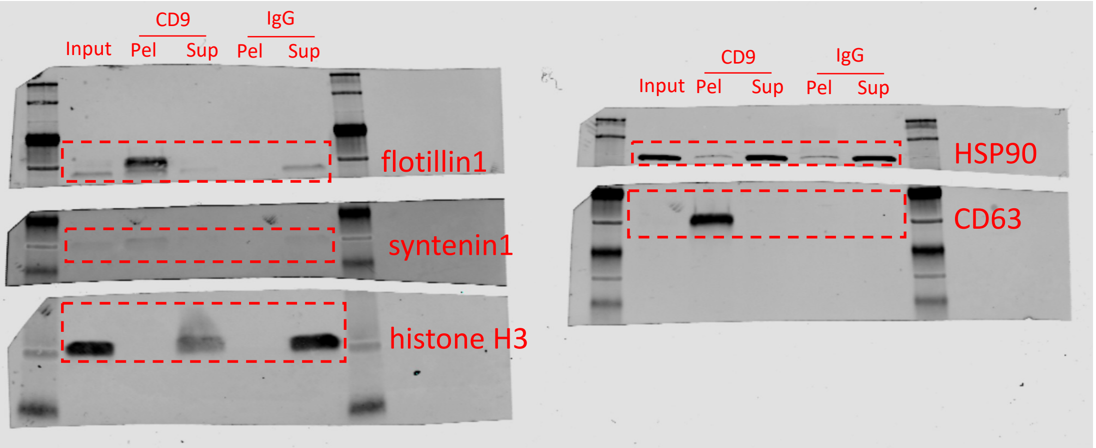

Supplement: Supplemental data [file jciinsight-7-159590-s205.pdf]
